# Encephalitis and poor neuronal death-mediated control of herpes simplex virus in human inherited RIPK3 deficiency

**DOI:** 10.1126/sciimmunol.ade2860

**Published:** 2023-04-21

**Authors:** Zhiyong Liu, Eduardo J. Garcia Reino, Oliver Harschnitz, Hongyan Guo, Yi-Hao Chan, Noopur Khobrekar, Mary L. Hasek, Kerry Dobbs, Darawan Rinchai, Marie Materna, Daniela Matuozzo, Danyel Lee, Paul Bastard, Jie Chen, Yoon Seung Lee, Seong K. Kim, Shuxiang Zhao, Param Amin, Lazaro Lorenzo, Yoann Seeleuthner, Remi Chevalier, Laure Mazzola, Claire Gay, Jean-Louis Stephan, Baptiste Milisavljevic, Soraya Boucherit, Flore Rozenberg, Rebeca Perez de Diego, Richard D. Dix, Nico Marr, Vivien Béziat, Aurelie Cobat, Mélodie Aubart, Laurent Abel, Stephane Chabrier, Gregory A. Smith, Luigi D. Notarangelo, Edward S. Mocarski, Lorenz Studer, Jean-Laurent Casanova, Shen-Ying Zhang

**Affiliations:** 1. St. Giles Laboratory of Human Genetics of Infectious Diseases, Rockefeller Branch, The Rockefeller University, New York, NY, USA.; 2. The Center for Stem Cell Biology, Sloan Kettering Institute for Cancer Research, New York, NY, USA.; 3. Human Technopole, Viale Rita Levi-Montalcini, Milan, Italy, EU.; 4. Department of Microbiology and Immunology, Emory Vaccine Center, Emory University, GA, USA.; 5. School of Medicine, Atlanta, GA, USA.; 6. Louisiana State University Health Sciences Center at Shreveport (LSUHSC-S), Shreveport, Louisiana, USA.; 7. Laboratory of Clinical Immunology and Microbiology, National Institute of Allergy and Infectious Diseases, NIH, Bethesda, MD, USA.; 8. Laboratory of Human Genetics of Infectious Diseases, Necker Branch, INSERM U1163, Necker Hospital for Sick Children, Paris, France, EU.; 9. Paris City University, Imagine Institute, Paris, France, EU.; 10. Pediatric Hematology-Immunology and Rheumatology Unit, Necker Hospital for Sick Children, AP-HP, Paris, France, EU; 11. Department of Pediatrics, CHU Saint-Etienne, Saint-Etienne, Paris, France, EU.; 12. Laboratory of Virology, Assistance Publique-Hôpitaux de Paris (AP-HP), Cochin Hospital, Paris, France, EU.; 13. Laboratory of Immunogenetics of Human Diseases, IdiPAZ Institute for Health Research, La Paz Hospital, Madrid, Spain, EU.; 14. Innate Immunity Group, IdiPAZ Institute for Health Research, La Paz Hospital, Madrid, Spain, EU.; 15. Interdepartmental Group of Immunodeficiencies, Madrid, Spain, EU.; 16. Viral Immunology Center, Department of Biology, Georgia State University, Atlanta, GA, USA.; 17. Department of Ophthalmology, Emory University School of Medicine, Atlanta, GA, USA.; 18. Research Branch, Sidra Medicine, Doha, Qatar.; 19. Institute of Translational Immunology, Brandenburg Medical School, Brandenburg an der Havel, Germany College of Health and Life Sciences, Hamad Bin Khalifa University, Doha, Qatar; 20. Pediatric Neurology Department, Necker Hospital for Sick Children, APHP, Paris-City University, France, EU.; 21. Department of Microbiology-Immunology, Northwestern University Feinberg School of Medicine, Chicago, IL, USA.; 22. Department of Pediatrics, Necker Hospital for Sick Children, Paris, France, EU.; 23. Howard Hughes Medical Institute, New York, USA.

## Abstract

Inborn errors of TLR3-dependent type I IFN immunity in cortical neurons underlie forebrain herpes simplex virus-1 (HSV-1) encephalitis (HSE) due to uncontrolled viral growth and subsequent cell death. We report an otherwise healthy patient with HSE who was compound heterozygous for nonsense (R422*) and frameshift (P493fs9*) *RIPK3* variants. Receptor interacting protein kinase 3 (RIPK3) is a ubiquitous cytoplasmic kinase regulating cell death outcomes, including apoptosis and necroptosis. *In vitro*, the R422* and P493fs9* RIPK3 proteins impaired cellular apoptosis and necroptosis upon TLR3, TLR4 or TNFR1 stimulation, and ZBP1/DAI-mediated necroptotic cell death following HSV-1 infection. The patient’s fibroblasts displayed no detectable RIPK3 expression. Following TNFR1 or TLR3 stimulation, the patient’s cells did not undergo apoptosis or necroptosis. Following HSV-1 infection, the cells supported excessive viral growth despite normal induction of antiviral IFN-β and interferon-stimulated genes (ISGs). This phenotype was, nevertheless, rescued by application of exogenous type I IFN. The patient’s human pluripotent stem cell (hPSC)-derived cortical neurons displayed impaired cell death and enhanced viral growth following HSV-1 infection, as did isogenic RIPK3-knockout hPSC-derived cortical neurons. Inherited RIPK3 deficiency therefore confers a predisposition to HSE, by impairing the cell death-dependent control of HSV-1 in cortical neurons independently of type I IFN immunity.

## One-sentence summary

Autosomal recessive RIPK3 deficiency impairs cell death-dependent control of HSV-1 in cortical neurons, underlying HSV-1 encephalitis.

## Introduction

Herpes simplex virus-1 (HSV-1) is an enveloped double-stranded DNA virus that infects most of the human population (1, 2). Infection is typically benign and limited to recurrent labial lesions, but this virus can cause life-threatening encephalitis in rare patients (3). HSV-1 encephalitis (HSE) occurs at a rate of ~2–4 cases per million inhabitants per year (4–8), corresponding to a prevalence of ~1–2 per 10,000 infected people worldwide. HSE is the most common sporadic (i.e. non-epidemic) encephalitis in the Western world. It can strike at any age, but there are two major peaks in incidence, the first at an age of six months to three years, corresponding essentially to disease following primary infection, and the second at an age of >50 years, probably following a viral reactivation event (7, 8). HSV-1 invades the central nervous system (CNS) via the olfactory bulb to cause forebrain herpes simplex encephalitis (HSE) (~95% of the patients) or, more rarely, via the trigeminal nerve to cause brainstem HSE (~5% of patients) (9, 10). The clinical manifestations depend on the location and extent of lesions, with presentations including fever, seizures, and altered consciousness (3). Remarkably, HSE is not accompanied by the dissemination of the virus to the blood and other organs. Patients do not even display the benign mucocutaneous labial lesions typically caused by HSV-1 in the general population. If untreated, mortality from this disease exceeds 70%. The advent of acyclovir in the 1980s decreased mortality rates to ~20% (11). Despite treatment, 40%-60% of survivors suffer from neurological sequelae, which are severe in about 10%-20% of cases (10, 12, 13).

HSV was first identified as the causal agent of HSE in 1941 (14), and HSV-1 was distinguished from HSV-2 in 1977 (15). The pathogenesis of HSE, apart from its viral etiology, long remained unexplained, as the virus remains in the peripheral sensory neural tissues in most infected individuals. Childhood HSE is no more common in children with inherited or acquired deficits of leukocytes, including a complete lack of T cells or B cells, or both, than in children with no such deficits (16). However, since 2006, autosomal recessive (AR) and autosomal dominant (AD) monogenic inborn errors of cell-intrinsic immunity have been identified as contributing to HSE susceptibility in children. Germline mutations of genes governing the TLR3 pathway (*TLR3*, *UNC93B1*, *TRIF*, *TRAF3*, *TBK1*, *IRF3, NEMO*) or the IFNAR1 pathway (*IFNAR1*, *TYK2*, *STAT1*, *STAT2*) have been implicated in this disease (17–29). The mechanism of HSE has been clarified through studies of human stem cell (hPSC)-derived peripheral and CNS cells. Mutations affecting the two connected pathways impair cortical neuron and oligodendrocyte cell-intrinsic type I IFN immunity to HSV-1 (30–32). TLR3 pathway gene mutations impair tonic and dsRNA-inducible levels of type I IFNs, whereas IFNAR1 pathway gene mutations impair cellular responses to type I IFNs. Mutations of *SNORA31* and *DBR1* have recently been shown to underlie forebrain HSE and brainstem viral encephalitis due to infection with HSV-1 or other viruses, respectively (33, 34). Overall, HSE appears to result from inborn errors of CNS-resident cell-intrinsic antiviral immunity in at least 5–10% of children with this rare disease. The clinical penetrance of these genetic defects is incomplete (8, 35), consistent with the sporadic nature of HSE (36). We hypothesized that additional unknown inborn errors of immunity might underlie HSE in other children.

## Results

### Biallelic predicted loss-of-function *RIPK3* mutations in a patient with HSE

**Figure 1.**
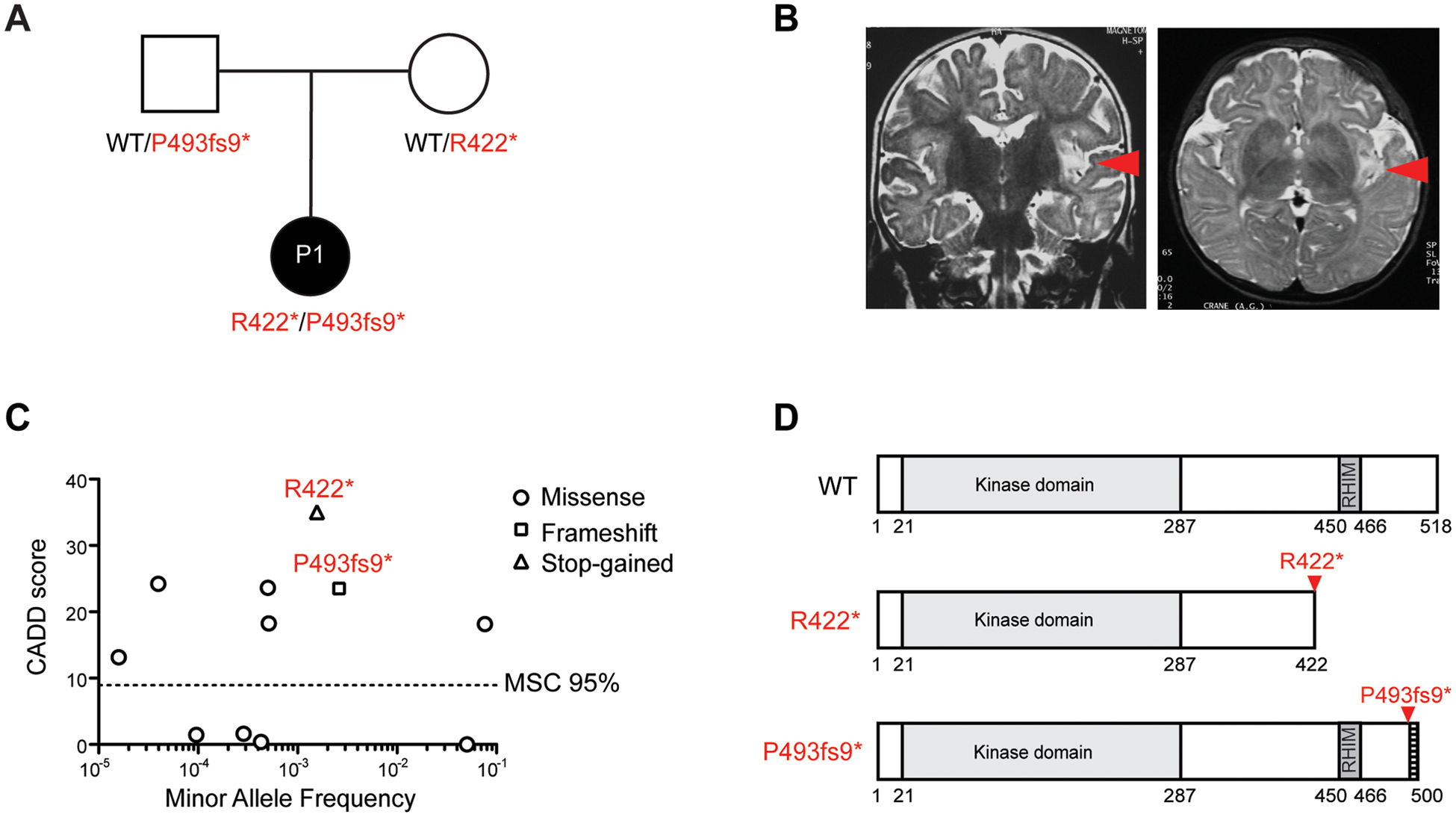
Compound heterozygous *RIPK3* mutations in a patient with HSE **A**. Family pedigree with allele segregation of the two *RIPK3* mutations. The proband (patient 1, P1), in black, is compound heterozygous for the p.Arg422* (R422*) and p. Pro493fs9* (P493fs9*) mutations. Each parent is heterozygous for one mutant allele. **B**. Images of the brain of P1, showing lesions affecting the left insula. **C**. Graph showing the CADD scores of all homozygous RIPK3 nonsynonymous or essential-splicing variants reported by the gnomAD database, and their minor allele frequency (MAF). MSC 95%: mutation significance cutoff for the 95% confidence interval. **D**. Schematic representation of the structure of the RIPK3 protein and the impact of the two mutations.

We studied a girl (patient 1, P1) born to healthy non-consanguineous parents originating from and living in France (fig. 1A). She was diagnosed with independent episodes of HSE at 6 and 17 months of age, and with autoimmune encephalitis (AE) one month after the second episode of HSE (fig. 1B, S1A,B and Supplementary clinical report). This girl was an only child. She had no history of any other severe infectious disease before or after these HSE episodes, despite having been infected with many other viruses in the past, as demonstrated by viral serology studies, and she remained otherwise healthy. Deep immunophenotyping by mass cytometry (CyTOF) revealed no obvious abnormalities in blood leukocyte subsets (fig. S1C). We performed whole-exome sequencing (WES) on the patient and both her parents. In P1, we searched for rare (minor allele frequency (MAF) < 0.01 in the Genome Aggregation Database (gnomAD) and in our in-house WES database containing ~15,000 exomes) non-synonymous or essential-splicing variants with a combined annotation-dependent depletion (CADD) score (37) higher than the mutation significance cutoff (MSC) (38), for genes with a gene damage index (GDI) below 13.83 (39). We considered *de novo* and biallelic variants, in accordance with the guidelines for single-patient genetic studies (40). We detected 20 *de novo* variants, none of which was deemed a plausible candidate based on biochemical nature or the expression of the corresponding genes or the function of their products (Table S1). This search also identified six genes for which P1 was homozygous or compound-heterozygous for variants (fig. S1D, Table S2), including two heterozygous mutations of *RIPK3* predicted to be loss-of-function (pLOF): p. Arg422* (c.1264 C>T, MAF 0.001568, CADD 35) and p. Pro493fs9* (c.1475 C>CC, MAF 0.002611, CADD 24.2). *RIPK3* encodes receptor-interacting protein kinase 3 (RIPK3). Sanger sequencing of DNA from the patient and her parents confirmed both mutations in the patient and showed that Arg422* (R422*) was inherited from the mother and Pro493fs9* (P493fs9*) from the father (fig. S1E).

### Population genetics of *RIPK3*

The human *RIPK3* gene is under modest negative selection, with a CoNeS of 0.8, consistent with an AR predisposition to life-threatening disease (fig. 1C, S1F). Only one homozygous carrier each was found for the R422* and P493fs9* variants among the 141,456 sequenced individuals from the gnomAD database (41). No other homozygous or compound heterozygous carrier of rare non-synonymous variants was found in our in-house exome database of data for ~15,000 patients with various infectious diseases. Only seven non-synonymous variants (MAF<0.01) were found in the homozygous state (11 homozygous carriers in total) in public databases (gnomAD and BRAVO, fig. 1C, Table S3). The rarity of homozygous carriers of these variants in the general population suggests that RIPK3 has an important function and is consistent with the low prevalence and incomplete penetrance of HSE. RIPK3 is a serine/threonine protein kinase involved in the extrinsic apoptosis and necroptosis death pathways (42–49) shown to be involved in host defense against herpesviruses, including HSV-1, both in cell culture and in mice (47, 50, 51). RIPK3 carries an N-terminal (N-ter) kinase domain and a C-terminal (C-ter) RIP homotypic interaction motif (RHIM) (fig. 1D), both of which are required for RIPK3-mediated necroptosis in response to various stimuli, including TNF and dsRNAs (52, 53). The RHIM is also required for RIPK3-mediated apoptosis (54). RIPK3 can homodimerize, oligomerize, or heterodimerize with RIPK1 via the RHIM domains of the two proteins, and the resulting RIPK3 homodimer and RIPK1/RIPK3 complex play different roles in the activation of necroptotic and apoptotic cell death pathways (54). The two pLOF variants of the patient are predicted to have different impacts on protein function, with R422* predicted to encode a protein with no RHIM domain, and P493fs9* predicted to encode a protein with an intact RHIM domain but lacking 26 C-terminal amino acids (a.a.), the impact of this deletion on RIPK3 function being unknown (fig. 1D). We hypothesized that compound heterozygosity for R422* and P493fs9* compromised RIPK3 function in this French child with recurrent HSE.

### Production of the R422* and P493fs9* RIPK3 proteins following transient transfection of HeLa and HEK293T cells

**Figure 2.**
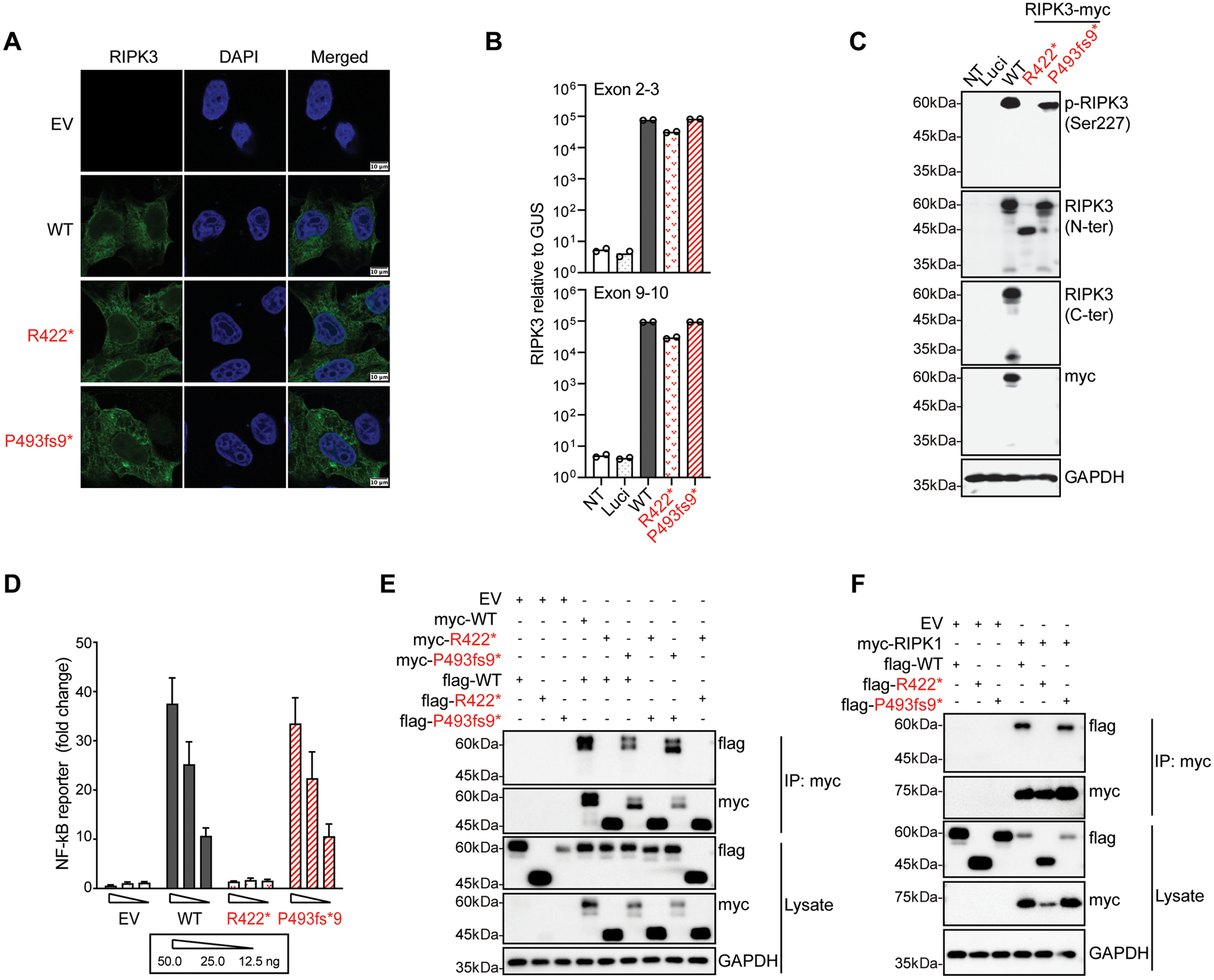
*In vitro* production and function of the RIPK3 variants upon transient transfection **A**. Confocal microscopy imaging of HeLa cells 24 h after transfection with wild-type (WT) and mutant *RIPK3*. Cells were stained with an antibody directed against the N-terminus (N-ter) of RIPK3 and the corresponding Alexa Fluor 488-conjugated secondary antibody (green). The nuclei were stained with DAPI (blue). Scale bar, 10 μm. The results shown are representative of three independent experiments. **B**. *RIPK3* mRNA levels were determined by reverse transcription-quantitative polymerase chain reaction (RT-qPCR) in HEK293T cells 24 h after transfection with a luciferase-expressing vector (Luci), or WT and mutant RIPK3 constructs. We used two probes, targeting exons 2–3 (upper panel) and exons 9–10 (lower panel) of *RIPK3*. The data shown are the means from two biological replicates from one experiment representative of three independent experiments. **C**. Immunoblotting analysis of total RIPK3 and autophosphorylated RIPK3 (p-RIPK3, Ser227) levels in HEK293T cells 24 h after transfection with C-terminally Myc-tagged RIPK3 WT and mutant constructs. RIPK3 proteins were detected with antibodies against the N terminus (N-ter) or C terminus (C-ter) of RIPK3, p-RIPK3 (Ser227) and Myc-tag. The results shown are representative of three independent experiments. **D**. RIPK3 overexpression-mediated NF-κB promoter-driven reporter assay in HEK293T cells, 24 h after transfection with the NF-κB reporter plasmid, along with various doses of empty vector (EV), WT and mutant RIPK3 constructs; analysis of luciferase activity. The data shown are the means of at least three biological replicates from three independent experiments. **E**. Myc-tagged WT and mutant RIPK3 constructs were co-expressed with FLAG-tagged WT and mutant RIPK3 constructs in HEK293T cells, which were then subjected to immunoprecipitation (IP) with anti-Myc antibody-conjugated agarose beads, and immunoblotting with anti-FLAG or anti-Myc antibodies. The results shown are representative of three independent experiments. **F**. HEK293T cells cotransfected with Myc-tagged RIPK1 and FLAG-tagged WT and mutant RIPK3 WT plasmids, subjected to IP and immunoblotting as in (**E**). The results shown are representative of three independent experiments.

RIPK3 is a nucleocytoplasmic protein located predominantly in the cytosol in the steady state (55, 56). We found that both the R422* and P493fs9* RIPK3 proteins were located in the cytoplasm, like the wild-type (WT) RIPK3, following plasmid-mediated transient expression in HeLa cells (fig. 2A). Similar levels of *RIPK3* mRNAs were detected following transient expression of C-ter (C-terminus) Myc-tagged R422*, P493fs9* and WT RIPK3 cDNAs in HEK293T cells, which have very low levels of endogenous RIPK3 (fig. 2B). Western blotting with an antibody (Ab) against N-ter (N-terminus) RIPK3, C-ter RIPK3 or Myc, detected the full-length WT RIPK3 as two bands at a molecular weight (MW) of ~60 kDa (fig. 2C). The lower band corresponds to the non-phosphorylated protein, as an antibody against the phosphorylated form (Ser227) of RIPK3 detected only the upper band (fig. 2C). In addition, bands were also detected at ~30 and ~25 kDa with the N-ter and C-ter Ab, respectively, suggesting that RIPK3 was cleaved upon overexpression (fig. 2C). Both the R422* and P493fs9* RIPK3 proteins were C-terminally truncated, as both were detected with the N-ter Ab but not with the C-ter Ab (fig. 2C). Moreover, R422* RIPK3 displayed impaired autophosphorylation and cleavage (fig. 2C).

### Function of the R422* and P493fs9* RIPK3 proteins following transient transfection of HEK293T cells

We investigated the activity of the mutant forms of RIPK3 by measuring NF-κB-dependent luciferase induction following the transient transfection of HEK293T cells. R422* was non-functional in this assay, whereas P493fs9* retained WT RIPK3-like activity (fig.2D, S2A and S2B). We also characterized all the other nine homozygous non-synonymous *RIPK3* variants from the gnomAD database in the same assay, seven of which had a MAF<0.01, the other two having a MAF between 0.01 and 0.1 (Table S3). One variant, Leu131Met (MAF = 0.0000398), was mildly hypomorphic, whereas the other eight variants were isomorphic (fig. S2C–E). The cumulative frequency of homozygotes for hypomorphic and LOF RIPK3 variants in the general population is, therefore, ~1.5E-5 (confidence interval: 3.8E-7 – 8.4E-5), a frequency consistent with the low prevalence and incomplete penetrance of HSE. RIPK3 has been shown to homodimerize or to heterodimerize with RIPK1 and TRIF via the RHIM domains of the two proteins (57–60). We therefore assessed the capacity of the R422* and P493fs9* variants to interact. P493fs9* was able to homodimerize, and to bind WT RIPK3, RIPK1 and TRIF, whereas R422* was not able to do so (Fig. 2E,F, S2F-H). This finding is consistent with the conservation in P493fs9* of an intact RHIM, which is clearly truncated in R422*, and the requirement of this motif for interaction with RHIM-containing proteins and the induction of NF-κB-dependent transcription (49, 61, 62). These data suggest that both the R422* and P493fs9* variants of RIPK3 are expressed as C-terminally truncated proteins, and that R422* is LOF for autophosphorylation, cleavage, NF-κB induction activity, and homo- or heterodimerization, whereas P493fs9* conserves these functions in overexpression conditions.

### Production of the mutant RIPK3 proteins following stable transduction of HT29 cells

**Figure 3.**
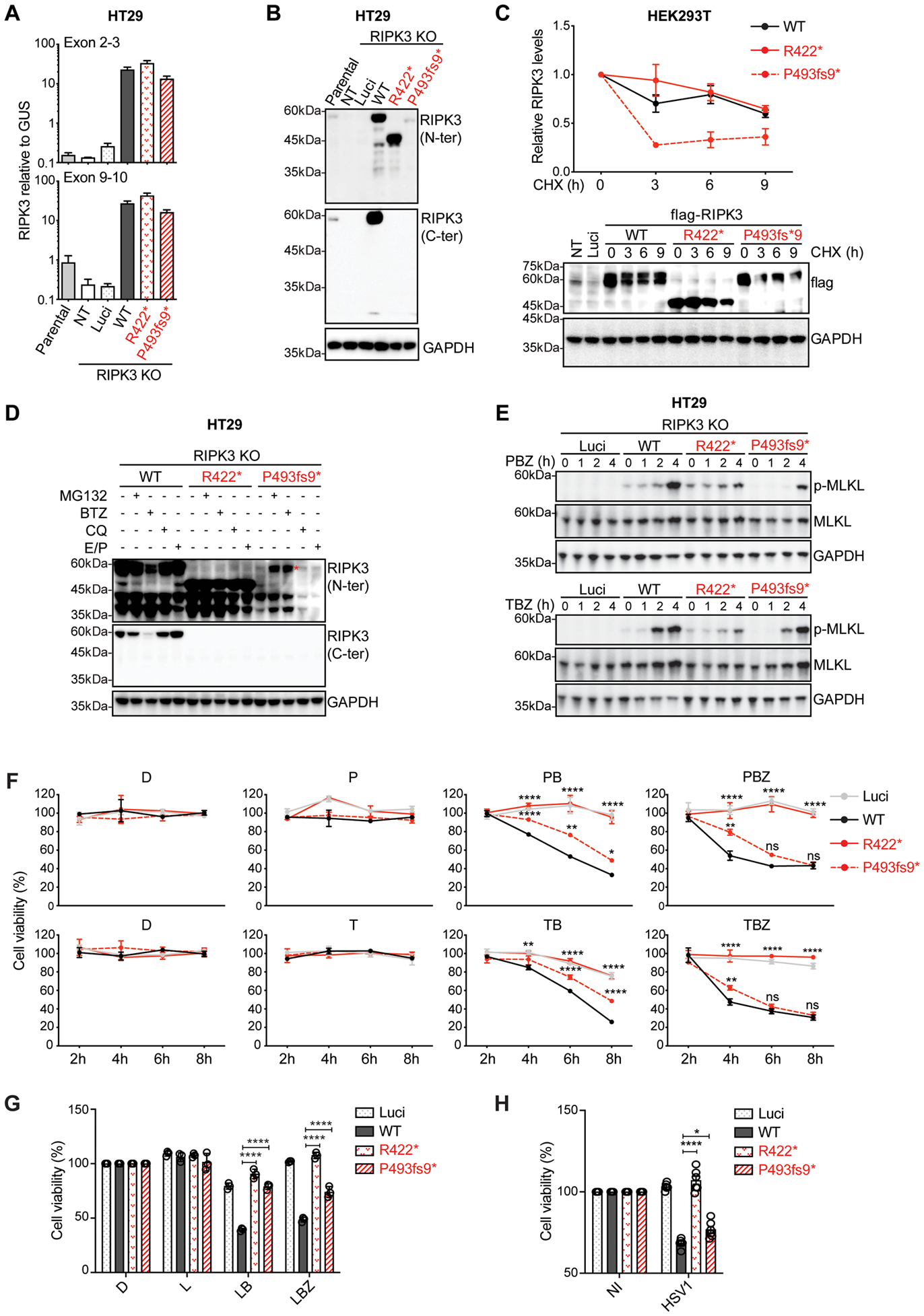
*In vitro* production and function of the RIPK3 variants after stable transduction **A**. *RIPK3* mRNA levels were determined by RT-qPCR in parental HT29 cells, or RIPK3 knockout (KO) HT29 cells left non-transfected (NT) or stably transfected with a mock vector (Luci) or with WT or mutant RIPK3 constructs in a lentiviral system. The data shown are the means from three biological replicates from three independent experiments. **B**. Immunoblotting analysis of RIPK3 protein levels in the parental HT29 cells, and in RIPK3 KO HT29 cells, as in (**A**). The results shown are representative of three independent experiments. **C**. Pulse-chase analysis to measure WT and mutant RIPK3 protein degradation. HEK293T cells were transfected with FLAG-tagged WT and mutant RIPK3 plasmids for 24 h. They were then treated with 100 μg/ml cycloheximide (CHX) for the indicated times, and subjected to western blotting (lower panel). The relative amounts of RIPK3 were calculated after normalization against GAPDH and are shown in the graph (upper panel). The results shown are representative of three independent experiments. **D**. Immunoblotting analysis of RIPK3 levels in RIPK3 KO HT29 cells stably expressing WT and mutant RIPK3, treated with protein degradation inhibitors (5 nM bortezomib (BTZ) for 12 h, 10 mM MG132, 50 mM chloroquine diphosphate (CQ), or 10 mg/mL E64d plus 10 mg/mL pepstatin) for 6 h. The red asterisk indicates the bands corresponding to RIPK3. The results shown are representative of three independent experiments. **E**. Immunoblot analysis of phosphorylated MLKL (p-MLKL, Ser358) in RIPK3 KO HT29 cells stably expressing Luci, WT or mutant RIPK3, treated with DMSO solvent as a control or with PBZ complex (consisting of poly(I:C), BV6 and Z-VAD), or TBZ complex (containing TNF, BV6 and Z-VAD) for the indicated times. The results shown are representative of three independent experiments. **F**. Viability of RIPK3^−/−^ HT29 cells stably expressing WT and mutant RIPK3 constructs, treated with DMSO solvent (control), or with poly(I:C), TNF, PB (poly(I:C) + BV6), TB (TNF + BV6), PBZ or TBZ complex for the indicated times. The results shown are the means ± SD from three biological replicates from one experiment, representative of three independent experiments. *P* values were obtained by one-way ANOVA and Tukey’s multiple comparison tests, and *P* values are indicated for the comparison of R422* or P493fs9* RIPK3 transduced cells with WT RIPK3 transduced cells. ns-not significant, *P<0.05, **P<0.01, ****P<0.0001. **G**. Viability of RIPK3^−/−^ HT29 cells stably expressing WT and mutant RIPK3 constructs, treated with DMSO solvent (control), or with L (LPS), LB (LPS + BV6) or LBZ (LPS + BV6 + Z-VAD) complex for 24 h. The results shown are the means ± SD from three biological replicates from one experiment, representative of two independent experiments. *P* values were obtained by one-way ANOVA and Tukey’s multiple comparison tests, and the corresponding *P* values are indicated. ****P<0.0001. **H**. Viability of RIPK3 KO HT29 cells stably co-expressing FLAG-tagged DAI with WT and mutant RIPK3, left non-infected (NI), or after infection with HSV-1 F*mut*RHIM (MOI=5) for 24 h. The data shown are means ± SEM from two independent experiments, with three biological replicates per experiment. *P* values were obtained by one-way ANOVA and Tukey’s multiple comparison tests, and the corresponding *P* values are indicated. *P<0.05, ****P<0.0001.

We analyzed the production of the R422* and P493fs9* RIPK3 proteins following the lentivirus-mediated transduction of RIPK3 knockout (KO) HT29 cells. The HT29 cell line is a human colorectal adenocarcinoma cell line with an epithelial morphology that is commonly used for cell death assays (48). Similar levels of RIPK3 mRNA were detected in cells stably transduced with R422*, P493fs9*, and WT RIPK3 (fig. 3A). Western blotting with the N-ter and C-ter RIPK3 antibodies revealed the presence of WT and mutant RIPK3 proteins, respectively, at molecular weights similar to those in HEK293T cells (fig. 3B, S3A). The autophosphorylation of endogenous RIPK3 and exogenous WT and mutant RIPK3 proteins was also detected in this system (fig. S3A). R422* and P493fs9* are C-terminally truncated proteins (fig. 3B, S3A), but only the P493fs9* protein was produced in smaller amounts than the WT RIPK3 (fig. 3B, S3A), suggesting that it may be poorly translated or subject to more intense posttranslational degradation. We compared the half-lives of the mutant and WT RIPK3 proteins in transiently transfected HEK293T cells following treatment with the protein synthesis inhibitor cycloheximide (CHX). The half-life of the P493fs9* protein was much shorter than those of R422* and WT RIPK3, suggesting that the P493fs9* protein is prone to degradation (fig. 3C). RIPK3 degradation has been reported to be mediated by both proteasome-dependent and lysosome-dependent pathways (63–65). We investigated both these degradation pathways in HT29 cells. P493fs9* mutant protein levels were rescued by treatment with proteasome inhibitors (MG132 and BTZ), but not by treatment with lysosomal protease inhibitors (CQ and E64d/Pep A) (fig. 3D). This result suggests that the P493fs9* variant is functionally isomorphic upon transient overexpression, but that the mutant protein is unstable and prone to excessive degradation. Thus, the mutant allele encoding this variant may be hypomorphic or LOF when expressed constitutively, given the low overall levels of the protein in the patient’s cells.

### Function of the mutant RIPK3 proteins in TLR3/4- or TNF-induced cell death in HT29 cells

We analyzed the function of the two mutant proteins following the stable transduction of RIPK3 KO HT29 cells (51). We first assessed the ability of these proteins to induce the phosphorylation of mixed-lineage kinase domain-like pseudokinase (MLKL), the main substrate of RIPK3 and the main known effector molecule of the necroptotic pathway (66, 67). Cells were treated with the pan-caspase inhibitor z-VAD, and the SMAC mimetic BV6, in combination with either the TLR3 agonist poly(I:C) (PBZ) or TNF (TBZ), to trigger necroptosis (43, 46, 48). Cells transduced with R422* or P493fs9* had lower levels of MLKL phosphorylation (p-MLKL) than RIPK3 KO HT29 cells transduced with the WT RIPK3 cDNA, after four hours of stimulation with PBZ (TLR3) or two hours of stimulation with TBZ (TNFR1) (fig. 3E, S3B and S3C). We also analyzed caspase 3 cleavage, a marker of apoptosis induction, upon TLR3 or TNFR1 stimulation with BV6 in the same cells, in combination with either poly(I:C) (PB) or TNF (TB). Caspase 3 cleavage levels were similar in the parental and RIPK3 KO HT29 cell lines (fig. S3D and S3E), suggesting that apoptosis was induced. We then assessed apoptosis- and necroptosis-mediated cell death upon TLR3, TLR4 or TNFR1 stimulation. Stimulation with poly(I:C), LPS or TNF, in the presence of BV6 alone (activating apoptotic signaling) or in the presence of both BV6 and z-VAD (necroptotic signaling), led to sustained high levels of cell death in HT29 RIPK3 KO cells transduced with WT RIPK3 (fig. 3F,G). By contrast, in R422*-transduced cells, the TLR3-, TLR4- or TNFR1-mediated induction of apoptotic or necroptotic cell death was abolished (fig 3F,G). P493fs9*-transduced cells displayed an impairment of the TLR3- or TNFR1-dependent induction of apoptotic or necroptotic cell death at early, but not late time points, as well as impaired but not abolished TLR4-mediated apoptotic or necroptotic cell death (fig. 3F), suggesting that this unstable variant is hypomorphic. As a control for RIPK1/3-independent apoptotic cell death, we used a combination of TNF and cycloheximide (TC) to stimulate the HT29 cells (68). As expected, similar levels of cell death were observed in RIPK3 KO HT29 cells transduced with empty vector, WT or mutant RIPK3 (Fig. S3F), confirming that RIPK3 had no effect on TC-induced apoptosis in HT29 cells, contrasting with its role in TLR3/4- and TNF-induced apoptotic or necroptotic cell death.

### Function of the mutant RIPK3 proteins in ZBP1/DAI-mediated cell death upon HSV-1 infection in HT29 cells

It has been shown in mice that ZBP1/DAI is a RHIM adaptor able to recruit RIPK3 to drive virus-induced necroptosis during infection with RHIM suppressor mutant herpesviruses (51, 69). We assessed the impact of our patient’s RIPK3 mutants on ZBP1/DAI-mediated necroptotic cell death in RIPK3 KO HT29 cells upon HSV-1 infection, comparing the results with those for WT RIPK3 and a previously reported kinase-dead RIPK3 mutant, D142N (70). Cell death rates were high in HT29 RIPK3 KO cells transduced with WT RIPK3, but not in those transduced with D142N RIPK3, as expected (fig. 3H, S3G–I). DAI-mediated necroptotic cell death was abolished in R422*-transduced cells and cell death rates in P493fs9*-transduced cells were lower than those in WT RIPK3-transduced cells (fig. 3H). Our data therefore suggest that, following the stable transduction of RIPK3 KO HT29 cells, the R422* and P493fs9* RIPK3 proteins abolish and impair, respectively, TLR3-, TLR4-, TNFR1-, and ZBP1/DAI-mediated, RIPK3-dependent apoptotic and necroptotic cell death. Compound heterozygosity for the two mutant alleles may, therefore, underlie a profound form of AR RIPK3 deficiency in the patient with HSE studied.

### Impaired RIPK3 protein production in the patient’s cells

**Figure 4.**
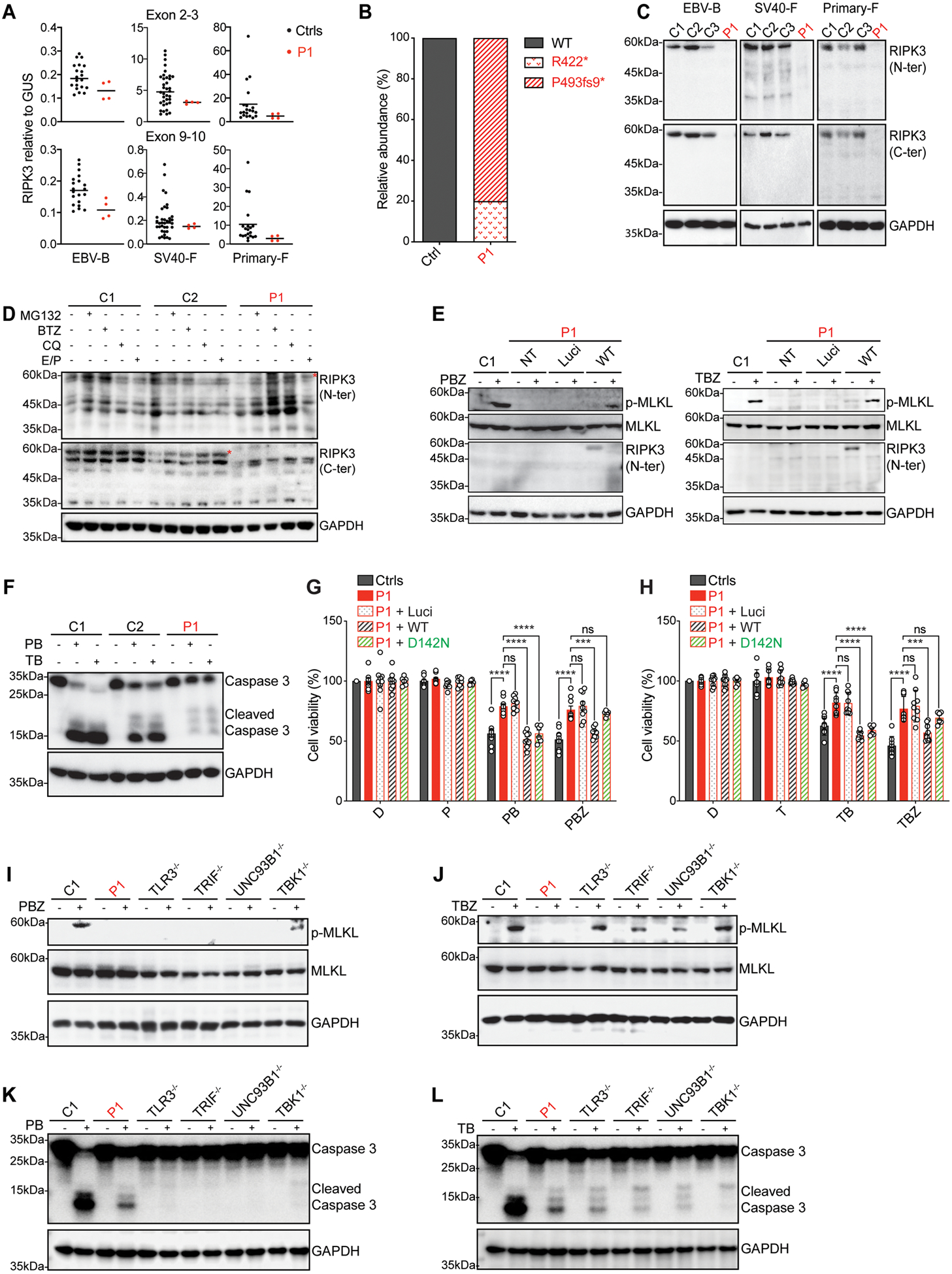
Impaired RIPK3 production and function in P1’s cells **A**. *RIPK3* mRNA levels were measured by RT-qPCR in EBV-B cells (EBV-B), SV40-fibroblasts (SV40-F) and primary fibroblasts (Primary-F) from healthy controls (Ctrls) and P1, with two probes targeting exons 2–3 (upper panel) and exons 9–10 (lower panel) of *RIPK3*. The results data shown are the means of four biological replicates from two independent experiments. **B**. Relative abundance (in percentages) of the RIPK3 cDNA generated from mRNA extracted from SV40-F from P1, assessed by TOPO-TA cloning. **C**. Immunoblot analysis of endogenous RIPK3 levels in EBV-B, SV40-F and Primary-F from healthy controls (C1, C2, C3) and P1, with antibodies against the N-terminus and C-terminus of RIPK3. The results shown are representative of more than three independent experiments. **D**. Immunoblot analysis of endogenous RIPK3 levels in SV40-F from healthy controls (C1, C2) and P1 treated with protein degradation inhibitors (5 nM BTZ for 12 h, 10 mM MG132, 50 mM CQ, or 10 mg/mL E64d plus 10 mg/mL pepstatin) for 6 h. The red asterisks indicates the bands corresponding to RIPK3. The results shown are representative of three independent experiments. **E**. Immunoblot analysis of p-MLKL levels in SV40-F from a healthy control (C1) and P1, either left non-transfected (NT) or transiently transfected with Luci or WT RIPK3 for 24 h, and then stimulated with PBZ or TBZ for 4 h. The red asterisks indicate the bands corresponding to RIPK3. The results shown are representative of three independent experiments. **F**. Immunoblot analysis of full-length and cleaved caspase 3 in SV40-F from healthy controls and P1, treated for 8 h with PB complex containing poly(I:C) and BV6, or TB complex containing TNF and BV6. The results shown are representative of three independent experiments. **G**. Viability of primary fibroblasts from healthy controls (Ctrls, *n*=3) and P1, either non- transduced or transduced with Luci, WT, variants with P1’s mutations or D142N RIPK3 lentiviruses for 48 h, then treated with DMSO solvent (D), or with poly(I:C), PB or PBZ complex for the times indicated. The results shown are the means ± SEM from three independent experiments (transduction with WT or P1 mutant RIPK3) and two independent experiments (for D142N RIPK3), with three biological replicates per experiment. **H**. Viability of primary fibroblasts from healthy controls (Ctrls, *n*=3) and P1, either non-transduced or transduced with Luci, WT, variants with P1’s mutations or D142N RIPK3 lentiviruses for 48 h, and treated with DMSO solvent (D), TNF, TB or TBZ complex for the times indicated. The results shown are the means ± SEM from three independent experiments (for WT or P1 mutant RIPK3) and two independent experiments (for D142N RIPK3), with three biological replicates per experiment. In **G and H,** *P* values were obtained by one-way ANOVA with Tukey’s multiple comparison tests, and the corresponding *P* values are indicated. ns-not significant, ***P<0.001, ****P<0.0001. **I-J**. Immunoblot analysis of p-MLKL in SV40-F from a healthy control (C1), P1, other HSE patients (TLR3^−/−^, TRIF^−/−^, UNC93B1^−/−^), and a TBK1^−/−^ patient, after stimulation with PBZ (**I**) or TBZ (**J**) for 4 h. The results shown are representative of three independent experiments. **K-L**. Immunoblot analysis of full-length and cleaved caspase 3 in SV40-F from a healthy control, P1, TLR3^−/−^, TRIF^−/−^, UNC93B1^−/−^, or TBK1^−/−^ patients, after stimulation with PB (**K**) or TB (**L**) for 8 h. The results shown are representative of three independent experiments.

The *RIPK3* mRNA is ubiquitously produced, in all tissues of the human body (https://proteinatlas.org/). Consistently, we detected RIPK3 mRNA in the various cell types studied, including Epstein-Barr virus-transformed B cells (EBV-B cells), SV40-T antigen-immortalized fibroblasts (SV40-fibroblasts), primary fibroblasts, and human pluripotent stem cell (hPSC)-derived cortical neurons (fig, S4A). However, RIPK3 mRNA levels were generally much lower in these cells than in the HT29 cell line, probably because RIPK3 production would be compromised by the culture of these human cells (48). We measured RIPK3 mRNA and protein levels in the patient’s cells. The total amounts of *RIPK3* mRNA in EBV-B cells, SV40-fibroblasts, and primary fibroblasts from P1 were towards the lower end of the range in healthy controls (fig. 4A). Moreover, the TOPO-cloning of cDNAs generated from mRNA from the patient’s SV40-fibroblasts revealed that ~80% of the *RIPK3* transcripts were P493fs9* transcripts, whereas only ~20% were R422* (fig. 4B), suggesting that R422* transcripts underwent nonsense-mediated mRNA decay. The RIPK3 protein was undetectable in EBV-B cells, SV40-fibroblasts, and primary fibroblasts from the patient when assessed with Abs recognizing the N-ter or C-ter of the protein (fig. 4C). In human fibroblasts heterozygous for the P493fs9* variant only, RIPK3 mRNA and protein levels were within the range of healthy controls (Fig. S4B, S4C), and only one band corresponding to WT RIPK3 protein was detected by western blotting with both N-ter and C-ter RIPK3 antibodies (Fig. S4C). Finally, following treatment of the patient’s SV40-fibroblasts with proteasome inhibitors (MG132 and BTZ) or lysosomal protease inhibitors (CQ and E64d/PepA), a band corresponding to the MW of the P493fs9* protein was detected by western blotting with an N-ter, but not a C-ter RIPK3 antibody (fig. 4D). Together with studies of individual alleles in HEK293T and HT29 cells, these findings suggest that RIPK3 proteins were undetectable in the cells of the patient due to the nonsense mRNA-mediated decay of R422* and the instability of the P493fs9* protein. These data suggest that compound heterozygosity for R422* and P493fs9* underlies a very profound, and perhaps complete, form of AR RIPK3 deficiency in this patient.

### Impaired RIPK3-dependent necroptotic and apoptotic cell death in the patient’s fibroblasts

RIPK3 is required for MLKL phosphorylation and the initiation of necroptosis downstream from TLR3/4, TNFR1, ZBP1/DAI, and IFNRs (71) in various cell types. We compared MLKL phosphorylation in SV40-fibroblasts from the patient with that in cells from healthy controls following stimulation with PBZ (TLR3) or TBZ (TNFR1). We detected p-MLKL clearly in control, but not in patient fibroblasts (fig. 4E and S4D). Transient plasmid-mediated exogenous WT RIPK3 expression rescued p-MLKL levels in the patient’s fibroblasts (fig. 4E), suggesting that compound heterozygosity for the two RIPK3 variants resulted in AR RIPK3 deficiency in these cells, accounting for resistance to necroptosis. Interestingly, by contrast to HT29 cells, in which RIPK3 KO did not affect caspase 3 cleavage in response to stimulation with PB or TB, caspase 3 cleavage in response to PB or TB was impaired in P1 fibroblasts (fig. 4F), suggesting that RIPK3 is directly or indirectly involved in TLR3- or TNFR1-mediated apoptosis in human fibroblasts. Primary fibroblasts from the patient were also more resistant than control cells to TLR3- and TNFR1-mediated necroptotic and apoptotic cell death (fig. 4G,H). Moreover, transient lentivirus-mediated exogenous WT RIPK3 expression rescued the sensitivity of the patient fibroblasts to TLR3- and TNFR1-mediated necroptotic and apoptotic cell death (fig. 4G,H). However, the kinase-dead mutant, RIPK3 D142N, was able to rescue TLR3- and TNFR1-mediated apoptotic cell death, but not necroptotic cell death in patient fibroblasts (fig. 4G,H and S4G,H), consistent with previous demonstrations of the dependence of RIPK3-mediated necroptosis, but not apoptosis, on the kinase activity of RIPK3 (48, 72, 73). Fibroblasts from other HSE patients with AR complete deficiencies of the TLR3 pathway (TLR3, TRIF, or UNC93B1) (19, 21, 28), serving as controls, displayed impaired TLR3-, but not TNFR1-mediated, necroptotic and apoptotic signaling (fig. 4I–L). By contrast, in fibroblasts from a patient with an AR deficiency of TBK1 (74) — a molecule at the crossroads of various antiviral pathways including TLR3 and functioning in parallel to IKBKE and RIPK1 (74) — necroptotic and apoptotic signaling upon TLR3 or TNFR1 stimulation was intact (fig. 4I–L). Overall, these findings suggest that AR RIPK3 deficiency in this patient has an overarching impact on known RIPK3 functions, as it profoundly impairs TLR3- and TNFR1-mediated necroptotic and apoptotic signaling, at least in fibroblasts.

### Normal TLR3- and TNFR1-dependent NF-κB and IRF3 pathways in the patient’s fibroblasts

**Figure 5.**
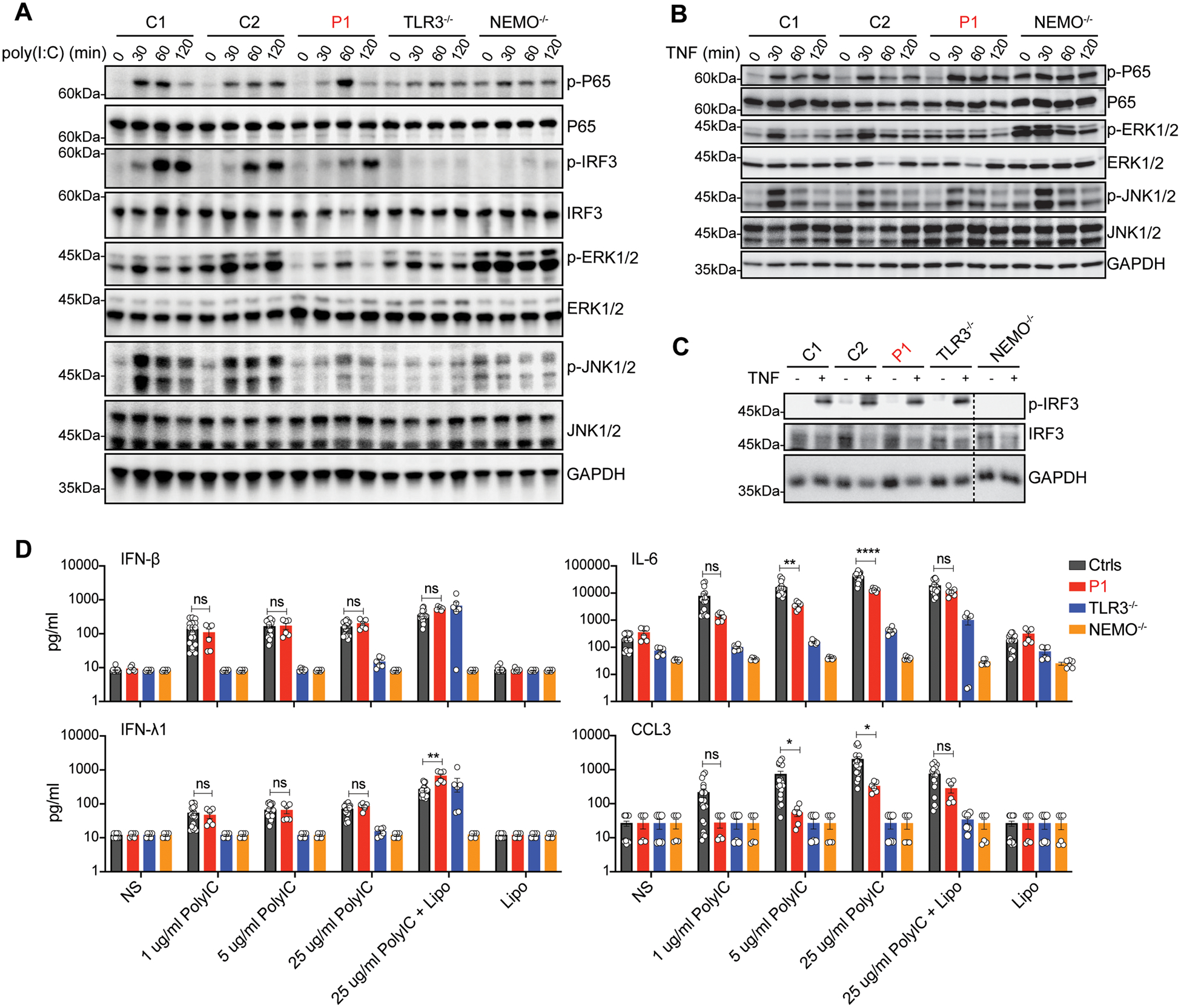
Intact signaling via the TLR3- and TNFR1-dependent NF-κB, IRF3 and MAPK pathways in P1 fibroblasts **A**. Immunoblot analysis of total and phosphorylated P65, IRF3, ERK1/2 and JNK1/2 in SV40-F from healthy controls (C1, C2), P1, TLR3^−/−^ and NEMO^−/−^ patients, after stimulation with 25 μg/ml poly(I:C) for the times indicated. The results shown are representative of three independent experiments. **B**. Immunoblot analysis of total and phosphorylated P65, ERK1/2 and JNK1/2 in SV40-F from healthy controls, P1 and a NEMO^−/−^ patient, after stimulation with 20 ng/ml TNF for the times indicated. The results shown are representative of three independent experiments. **C**. Immunoblot analysis of total and phosphorylated IRF3 in SV40-F from healthy controls, P1, TLR3^−/−^ and NEMO^−/−^ patients, after stimulation with 20 ng/ml TNF for 24 h. The results shown are representative of three independent experiments. **D**. SV40-F from healthy controls (Ctrls, *n*=3), P1 and TLR3^−/−^ and NEMO^−/−^ HSE patients were left unstimulated (NS) or were stimulated with various doses of poly(I:C) alone, Lipofectamine alone (Lipo), or both (poly(I:C)+Lipo), for 24 h. The amounts of IFN-β, IFN-λ1, IL-6 and CCL3 in culture supernatants were determined with Legendplex cytometric bead arrays. The results shown are the means ± SEM from two independent experiments, with three biological replicates per experiment. Each dot represents one biological replicate. *P* values were obtained by one-way ANOVA with Tukey’s multiple comparison tests, and the corresponding *P* values are indicated. ns-not significant, *P<0.05, **P<0.01, ****P<0.0001.

RIPK3 has been implicated in cell death-independent signal transduction in addition to the necroptotic and apoptotic signaling pathways (75). It acts particularly through the recruitment of RIPK1, and TRIF (60), via their RHIMs. We compared the activation of NF-κB, IRF3 and MAPKs following the stimulation of TLR3 and TNFR1, between fibroblasts from P1 and from healthy controls and other patients with recessive, complete TLR3 or NEMO deficiency. Following endosomal stimulation with poly(I:C), the activation of P65 and IRF3 was normal in SV40-fibroblasts from P1, with ERK1/2 and JNK1/2 activation only mildly decreased (not abolished), whereas the phosphorylation of P65, IRF3, and MAPKs (including ERK1/2, JNK1/2) was completely abolished in fibroblasts from TLR3- or NEMO-deficient patients (fig. 5A). Following stimulation with TNF, the activation of NF-κB, IRF3 and MAPKs was intact in fibroblasts from P1 and TLR3-deficient patients (fig. 5B–C). In NEMO-deficient cells, which served as a negative control in these experiments (76, 77), the activation of P65, ERK1/2 and IRF3 upon stimulation with poly(I:C) or TNF was abolished. Thus, RIPK3 deficiency had a modest impact on the TLR3-mediated activation of NF-κB, IRF3 and MAPKs, whereas the activation of TNFR1-mediated signaling was intact. These findings suggest that RIPK3 is redundant for the activation of these pathways, at least in fibroblasts. The patient’s cells would therefore probably display normal type I IFN production following the stimulation of TLR3 by dsRNA (including normal baseline IFN-β levels), and during the course of viral infection.

### Normal TLR3-dependent induction of anti-viral IFNs in the patient’s fibroblasts

We measured the production of IFNs and other cytokines in RIPK3-deficient fibroblasts from P1 following TLR3 activation by poly(I:C). Interestingly, unlike TLR3- and NEMO-deficient fibroblasts, in which the TLR3-mediated production of antiviral IFN-β, IFN-λ and other cytokines and chemokines was impaired (fig. 5D and S5A), only the TLR3-mediated production of IL-6, IL-8 and CCL3 was impaired in fibroblasts from P1, which had normal levels of production for IFN-β, IFN-λ, and the other cytokines tested (fig. 5D and S5A). Similar results were obtained in assessments of mRNA levels for *IFNB*, *IFNL,* and ISGs (*MX1*, *IFIT1*, *ISG15*) by RT-qPCR in the same cells following TLR3 activation (fig. S5B). Consistent with the intact TNFR1-mediated activation of NF-κB, IRF3 and MAPKs in P1’s fibroblasts, the production of IFNs and of the other cytokines tested was similar in fibroblasts from P1 and in those from controls, following TNF stimulation (fig. S5C). Moreover, the patient’s fibroblasts displayed a normal response to IFN-α2b, as shown by normal mRNA induction for ISGs including *IFIT1* and *MX1* (fig. S5D), indicating that the signaling of the IFNAR1-mediated type I IFN response pathway was intact. Together, these data suggest that RIPK3 is important for the TLR3-mediated production of a few specific proinflammatory cytokines and chemokines, such as IL6, IL-8 and CCL3, probably related to the mild decrease in ERK1/2 and JNK1/2 activation by poly(I:C) stimulation (fig. 5A). However, RIPK3 is largely redundant, at least in fibroblasts, for the production of antiviral type I and III IFNs and other cytokines. A role of impaired TLR3-dependent chemokine production in the pathogenesis of HSE in P1 cannot be excluded, as a previous study suggested that RIPK3 may coordinate immune responses by mediating chemokine production in West Nile virus-infected mouse neurons (78). However, the molecular mechanisms by which human RIPK3 deficiency underlies HSE do not involve an impairment of type I IFN-mediated antiviral immunity, contrasting with the situation in patients with HSE due to mutations of the TLR3-IFNAR1 circuit. The pathogenesis of HSE due to RIPK3 deficiency is probably related to an impairment of TLR3-, and/or ZBP1/DAI-mediated, and perhaps TNFR1-mediated, necroptotic and apoptotic signaling.

### Enhanced HSV-1 replication in the patient’s fibroblasts

**Figure 6.**
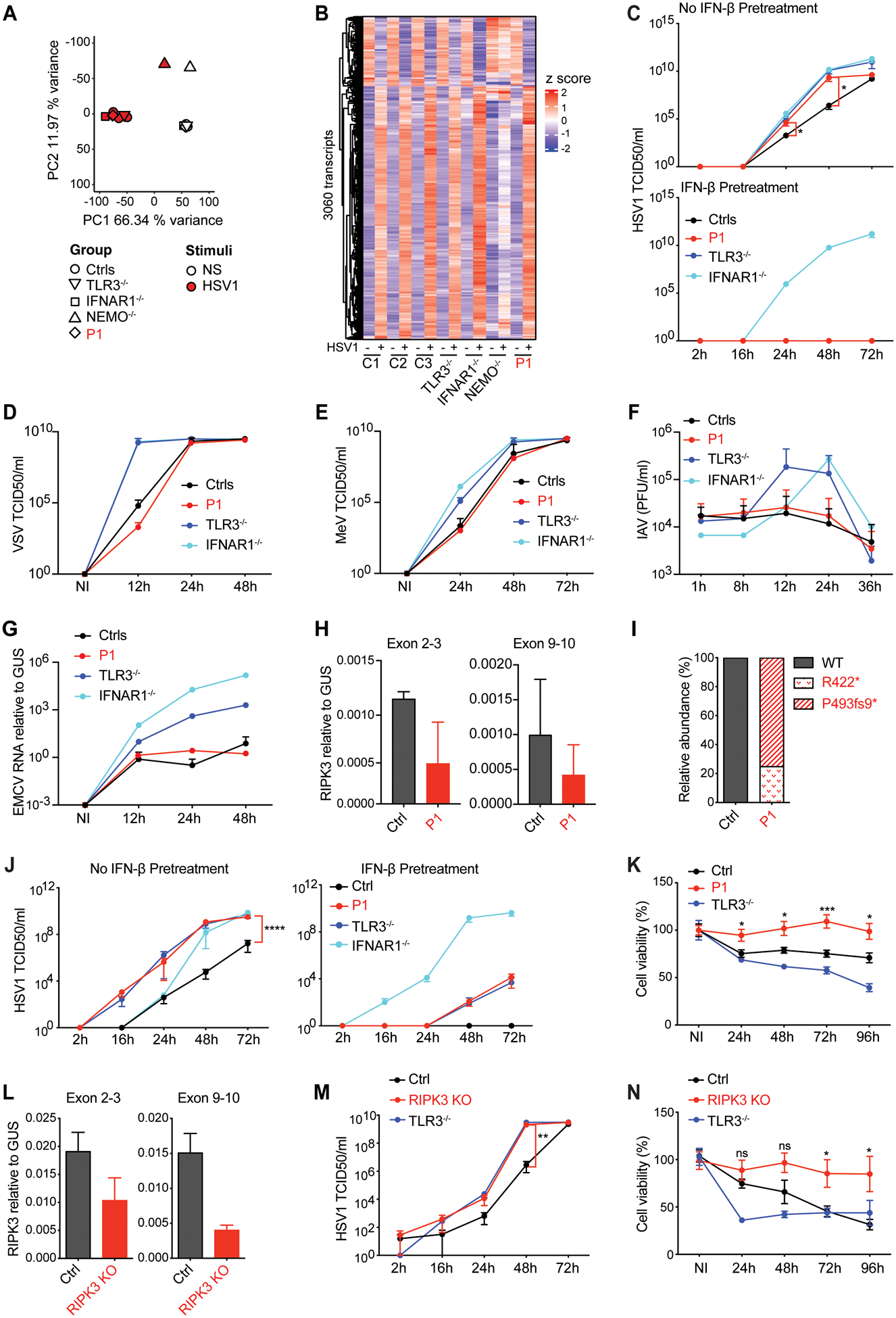
Enhanced susceptibility of *RIPK3*-deficient fibroblasts and hPSC-derived cortical neurons to HSV-1 **A**. Principal component analysis of the transcriptome of human primary fibroblasts without (NS) or with HSV-1 infection for 24 hours, in cells from healthy controls (Ctrls, *n*=3), P1 and other patients with recessive TLR3, IFNAR1 or NEMO deficiency. **B**. Genes differentially expressed between HSV-1 and NS in human primary fibroblasts, as in (**A)**. Heatmap including 3060 genes with relative absolute fold-changes in expression > 2 (in all three healthy controls) in response to HSV-1 relative to NS samples. **C**. SV40-F from healthy controls (Ctrls, *n*=3), P1 and other patients with recessive TLR3, IFNAR1 or NEMO deficiencies were left untreated or were treated with IFN-β for 24 h, and then infected with HSV-1 (MOI = 0.001). Virus replication was then evaluated at the indicated timepoints post infection. HSV-1 replication was quantified by the TCID_50_ virus titration method. The data shown are the means ± SEM of two independent experiments with two biological replicates per experiment. P values were obtained by one-way ANOVA with Tukey’s multiple comparison tests, and the *P* values for the 24 h and 48 h time points are indicated for the comparison of P1’s cells with control cells. *P<0.05. **D-G**, Virus replication levels for VSV (**D**), measles virus (MeV) (**E**), influenza A virus (IAV) (**F**), and EMCV (**G**) in SV40-F, as in (**C**), at the indicated times post infection with VSV (MOI = 0.1), MeV (MOI = 0.5), IAV (MOI = 10), or EMCV (MOI = 0.01), as assessed by the TCID_50_ virus titration method (VSV and MeV), plaque assay (IAV), or expression levels of the three-dimensional region of the EMCV genome, as measured by RT-qPCR. The data shown are the means of two to four biological replicates from two (IAV and EMCV) or four (VSV, MeV) independent experiments. **H**. *RIPK3* mRNA levels, as measured by RT-qPCR, in cortical neurons differentiated from the hPSCs of healthy controls and P1. We used two probes, targeting exons 2–3 (left) and exons 9–10 (right) of *RIPK3*. The data shown are the means of two biological replicates from one experiment. **I**. Relative abundance (in percentages) of the RIPK3 cDNA generated from mRNA extracted from hPSC-derived cortical neurons from a healthy control and P1, assessed by TOPO-TA cloning. **J**. hPSC-derived cortical neurons from a healthy control, P1 and other HSE patients with AR TLR3 or IFNAR1 deficiencies, with or without IFN-β pretreatment for 24 h, were infected with HSV-1 (MOI=0.001) and virus replication levels were measured at the indicated timepoints post infection. HSV-1 replication was quantified by the TCID_50_ virus titration method. The data shown are means ± SEM for four independent experiments (a healthy control, P1 and TLR3-deficienct cells) and two independent experiments (IFNAR1-deficiency cells). *P* values were obtained by one-way ANOVA with Tukey’s multiple comparison tests, and the *P* values for the 72 h time points are indicated for the comparison of P1’s cells with control cells. ****P<0.0001. **K**. Viability of hPSC-derived cortical neurons from a healthy control, P1 and a TLR3^−/−^ HSE patient, left non-infected (NI), or infected with HSV-1 (MOI=0.001) for the times indicated. The data shown are means ± SEM for two independent experiments, with three biological replicates per experiment. *P* values were obtained by one-way ANOVA with Tukey’s multiple comparison of P1’s cells with control cells, and the corresponding *P* values are indicated. *P<0.05, ***P<0.001. **L**. *RIPK3* mRNA levels, as measured by RT-qPCR, in cortical neurons from parental and *RIPK3* KO hPSCs. We used two probes, targeting exons 2–3 (left) and exons 9–10 (right) of *RIPK3*. The data shown are the means of three biological replicates from one experiment. **M**. hPSC-derived cortical neurons derived from parental healthy control cells, *RIPK3* KO cells and HSE patients with AR TLR3 were infected with HSV-1 (MOI=0.001) for the times indicated. HSV-1 replication was quantified by the TCID_50_ virus titration method. The data shown are means ± SEM for three independent experiments (the parental cells and RIPK3 KO cells) and two independent experiments (TLR3-deficient cells). P values were obtained by one-way ANOVA with Tukey’s multiple comparison tests, and the *P* value for the 48 h time point is indicated for the comparison of RIPK3 KO cells with control parental cells. **P<0.01. **N**. Viability of hPSC-derived cortical neurons from healthy parental control cells, *RIPK3* KO cells and a TLR3^−/−^ HSE patient, left non-infected, or infected with HSV-1 (MOI=0.001) for the times indicated. The data shown are means ± SEM for two independent experiments, with three biological replicates per experiment. P values were obtained by one-way ANOVA with Tukey’s multiple comparison tests, and the *P* values for the indicated timepoints are indicated for the comparison of RIPK3 KO cells with control parental cells. The corresponding *P* values are indicated. ns - not significant, *P<0.05.

We have shown that HSE-causing TLR3 pathway mutations impair TLR3-dependent type I IFN-mediated cell-intrinsic immunity to HSV-1 in fibroblasts and hPSC-derived cortical neurons (20). We tested SV40-fibroblasts, which have been shown to be a surrogate cell type for studies of type I IFN-dependent and -independent cell-intrinsic immunity to viruses, including HSV-1 (19, 20, 23–27). Consistent with the normal production of IFN-β and IFN-λ by P1’s SV40-fibroblasts upon TLR3 stimulation (fig. 5D and S5A–B), HSV-1 infection (KOS strain) of the patient’s SV40-fibroblasts also induced normal levels of *IFNB* and *IFNL* production, similar to those in healthy controls (fig. S6A). Furthermore, the cellular responses to HSV-1, as assessed by bulk RNA sequencing (RNA-seq) on primary fibroblasts from the patient, were normal at genome-wide transcriptome level (Fig. 6A,B), including for the induction of all fibroblastic ISGs (32) assessed (Fig. S6B), as shown by comparisons with cells from healthy controls. Nevertheless, P1’s fibroblasts displayed enhanced HSV-1 replication at various time points after HSV-1 infection at a low multiplicity of infection (MOI=0.001), in multiple cycle conditions (fig. 6C), at levels similar to those in TLR3-deficient or IFNAR1-deficient fibroblasts from other HSE patients, but higher than those in healthy controls. Exogenous IFN-β pretreatment rendered P1’s fibroblasts and TLR3-deficient fibroblasts resistant to HSV-1 infection, but had no such effect on IFNAR1-deficient fibroblasts (fig. 6C). Finally, unlike fibroblasts from AR TLR3- or IFNAR1-deficient patients, P1’s fibroblasts displayed normal control of infection for the other neurotropic viruses tested, including vesicular stomatitis virus (VSV), measles virus (MeV), encephalomyocarditis virus (EMCV) and influenza A virus (IAV), similar to that in cells from healthy controls (Fig. 6D–G). Therefore, despite their intact type I IFN antiviral immunity, P1’s fibroblasts are specifically highly susceptible to HSV-1, probably due to impaired control of viral growth via necroptotic/apoptotic cell death, which plays a particularly important role in the control of HSV-1 infection.

### Low levels of cell death and enhanced HSV-1 replication in the patient’s cortical neurons

We have shown that HSE-causing mutations impair cell-intrinsic immunity to HSV-1 in hPSC-derived CNS cortical neurons and oligodendrocytes due to the disruption of TLR3-dependent type I IFN-mediated antiviral immunity (mutations of the TLR3-IFNAR1 circuit) (30) or new antiviral mechanisms (mutations of *SNORA31*) (34). Programmed cell death pathways are key mechanisms in host antiviral defenses, particularly against viruses that invade the central nervous system (79). We tested CNS neurons from patient-specific hPSCs reprogrammed from P1’s primary fibroblasts (fig. S6C–E). *RIPK3* mRNA levels were low in P1’s neurons (fig. 6H), consistent with the results obtained for P1’s EBV-B cells and fibroblasts. TOPO-cloning of the cDNA generated from the mRNA of the patient’s hPSC-derived neurons revealed that ~80% of the *RIPK3* transcripts were P493fs9*, whereas only ~20% were R422* (fig. 6I), as in fibroblasts, suggesting that R422* mRNA also underwent nonsense-mediated decay in cortical neurons. HSV-1 replication levels were much higher in hPSC-derived cortical neurons from P1 than in those from healthy controls, as were those in TLR3- and IFNAR1-deficient hPSC-derived neurons, at various times points after infection with HSV-1 at a MOI of 0.001 (fig. 6J). As in P1’s fibroblasts, enhanced HSV-1 replication was rescued by pretreatment with exogenous IFN-β in P1’s and TLR3-deficient hPSC-derived neurons, but not in IFNAR1-deficient hPSC-derived neurons (fig. 6J). However, unlike TLR3-deficient neurons, which had higher levels of virus-induced cell death following HSV-1 infection, neurons from P1 displayed enhanced resistance to HSV-1 infection-induced cell death, even relative to healthy control hPSC-derived neurons (fig. 6K). These data suggest that RIPK3-deficient cortical neurons are highly susceptible to HSV-1 due to defective HSV-1-induced necroptotic and apoptotic cell death-dependent antiviral defenses.

### Enhanced HSV-1 replication and low levels of cell death in RIPK3 KO cortical neurons

Finally, as a means of establishing a causal relationship between genotype (RIPK3 deficiency) and phenotype (low levels of cell death-dependent antiviral immunity resulting in enhanced HSV-1 growth), we generated CRISPR/Cas9-mediated RIPK3 knockout (KO) hPSC lines, which were differentiated into cortical neurons (fig. S6F–I). The relative levels of *RIPK3* mRNA in RIPK3 KO neurons were low, suggesting that transcripts were destroyed by nonsense-mediated mRNA decay (fig. 6L). RIPK3 KO neurons, like hPSC-derived neurons from P1, had much higher levels of HSV-1 than the parental WT cells, a phenotype that was rescued by IFN-β pretreatment (fig. 6M and S6J). Like hPSC-derived neurons from P1, RIPK3 KO neurons also displayed enhanced resistance to HSV-1-induced cell death (fig. 6N). Together, these data confirm that impaired RIPK3-dependent apoptotic and/or necroptotic cell death signaling-mediated antiviral immunity renders RIPK3-deficient hPSC-derived cortical neurons prone to HSV-1 infection. The activation of apoptotic and necroptotic cell death pathways during HSV-1 infection appears to depend on TLR3 and/or ZBP1/DAI, which are controlled by RIPK3, although a role for other antiviral sensors (e.g. TNFR1) cannot be excluded. Our data provide a plausible molecular and cellular mechanism of HSE in this patient with AR RIPK3 deficiency.

## Discussion

AR RIPK3 deficiency is a new genetic etiology of childhood HSE. Approximately 5% of the children studied in our international HSE cohort have experimentally proven AR or AD deficiencies of the TLR3-IFN-α/β circuit, which governs cell-intrinsic immunity in specific organs of the human body, including the brain and the lung (80–82), and another ~2% have AD or AR deficiencies of snoRNA31 or DBR1, which govern new antiviral mechanisms that appear to be specific to the forebrain and brainstem, respectively (33, 34). TLR3-induced apoptotic and necroptotic signaling is impaired in RIPK3-deficient fibroblasts, resulting in impaired TLR3-dependent apoptotic and necroptotic cell death. TLR3-dependent apoptotic and necroptotic signaling is also impaired in fibroblasts from other HSE patients with deleterious mutations of *TLR3* or of some of the genes encoding components of its signaling pathway (*UNC93B1*, *TRIF*). Impaired TLR3-RIPK3-dependent apoptotic and necroptotic cell death-mediated antiviral immunity may, therefore, have played a role in HSE pathogenesis in the patient with AR RIPK3 deficiency studied here, and in other patients with deficiencies of the TLR3 pathway. However, in HSE patients with inborn errors of the TLR3 pathway, the TLR3-dependent production of IFN-α/β, -λ and of many other cytokines was also impaired, whereas RIPK3 deficiency impaired the production of only a narrow range of cytokines, not including antiviral IFNs. Moreover, RIPK3 deficiency impaired apoptotic and necroptotic cell death signaling not only via TLR3, but also via ZBP1/DAI, the proposed sensor inducing HSV-1-induced necroptotic cell death (51). It also affects cell death signaling via other sensors, such as TLR4 and TNFR1. RIPK3-deficient hPSC-derived cortical neurons were resistant to HSV-1-induced cell death, leading to excessive virus replication, whereas TLR3-deficient neurons underwent HSV-1-induced cell death earlier, due to enhanced viral replication. Inborn errors of RIPK3 and the TLR3-IFN circuit may, thus, lead to both common and specific molecular mechanisms of disease, impairing cell-intrinsic antiviral immunity in an overlapping manner.

*In vitro* studies have shown that the RIPK1/RIPK3 complex may be involved in regulating virus-induced cell necroptosis, which can be triggered by various viral agents, including influenza A virus (IAV) (44), RHIM suppressor mutants of murine cytomegalovirus (HCMV and MCMV) (45), or herpes simplex viruses (HSV-1 and HSV-2) (46, 47), and E3-deficient vaccinia virus (43). A role for RIPK3-mediated cell death in host antiviral defense was first proposed in 2009, following studies of vaccinia virus infection *in vivo* (43). Ripk3-deficient mice also display impaired HSV-1-induced necrosis, and uncontrolled HSV-1 replication and pathogenesis in various organs, including the brain (50). Meanwhile, Ripk3-deficient mice displayed levels of murine gammaherpesvirus 68 (MHV68) replication similar to that in the WT mice, at least in the lung and the spleen (83), suggesting that the role of RIPK3 in host antiviral immunity might be virus- or organ-specific. In addition to the contribution of RIPK3-mediated cell death to restricting viral pathogenesis, cell death-independent functions of RIPK3 may also specifically limit viral invasion of the CNS. Ripk3-deficient mice are susceptible to CNS infection by West Nile virus (WNV) and Zika virus (ZIKV) *in vivo* (78, 84). Interestingly, it has been suggested that the enhanced susceptibility of Ripk3^−/−^ mice to WNV is due to an abolition of neuronal chemokine expression, leading to lower levels of T-lymphocyte and myeloid-cell recruitment to the CNS to restrict viral infection (78). In another study, an enhanced viral load in Ripk3^−/−^ mouse brain following infection with ZIKV was attributed to impaired activation of the nucleotide sensor ZBP1 and downstream RIPK1 and RIPK3 signaling to restrict viral replication, due to changes in cellular metabolism due to the upregulation of an enzyme, IRG1, and production of the metabolite itaconate (84). RIPK3 therefore seems to control mouse cortical neuron-intrinsic immunity to viruses through canonical cell apoptosis and necroptosis pathways and non-canonical functions (78, 84). In this study, we observed impaired TLR3-mediated CCL3 production in RIPK3-deficient fibroblasts from our patient, but the pathophysiological role of this impairment of CCL3 production remains unclear.

Remarkably, the RIPK3-deficient patient, who is now 24 years old, has not suffered from any severe infectious disease other than HSE, viral or otherwise, despite infection with many common viruses (fig. S1A,B). Human RIPK3 may be crucial to protect the CNS against HSV-1 through control over cortical neuron cell-intrinsic immunity to HSV-1, but otherwise largely redundant (85). This hypothesis is supported by our *in vitro* data showing that RIPK3-deficient fibroblasts from our patient are susceptible to HSV-1, but able to control the other four viruses tested normally. This is also reminiscent of the normal clearance of MHV68 in Ripk3-deficient mice (83). More patients are required to delineate more accurately the range of infections and other phenotypes associated with this deficiency (85). However, the clinical phenotype of this patient already contrasts sharply with that of inherited RIPK1 deficiency (86, 87). Fourteen patients with AR RIPK1 deficiency, which became symptomatic between the ages of one day and six months and was diagnosed between the ages of one day and 11 years, have been reported (86–88). The main clinical phenotypes of these RIPK1-deficient patients include early-onset inflammatory bowel disease, polyarthritis, and recurrent viral, bacterial, and fungal infections. Viruses as diverse as human cytomegalovirus (HCMV), varicella zoster virus (VZV), respiratory syncytial virus (RSV), and HSV-1 have been reported to underlie severe infections of the skin and digestive and respiratory mucosae. These patients also displayed multiple leukocytic abnormalities, including profound and broad NK, T, and B lymphopenia. The cellular and molecular basis of viral disease in RIPK1 deficiency has yet to be studied, and it is unclear whether RIPK1 deficiency affects non-leukocytic, cell-intrinsic immunity to viruses. HSV-1 infection status is unknown for most patients. However, given their very young age at diagnosis, these patients are unlikely to have been infected with HSV-1 before IgG substitution or hematopoietic stem cell transplantation. In such circumstances, we would predict that RIPK1-deficient patients would probably be prone to HSE. By contrast, the absence of obvious leukocytic abnormalities in the RIPK3-deficient patient is consistent with the absence of serious infections other than HSE in this patient until the age of 24 years. Future studies searching for inborn errors of RIPK1, RIPK3, and related molecules in patients with HSE and other diseases will improve our understanding of RIPK1 and RIPK3 biology in natural conditions (89–92).

## Materials and methods

### Study design

We first performed whole-exome sequencing (WES) on one patient who had recurrent Herpes simplex virus 1 (HSV-1) encephalitis (HSE) during her childhood, and both her parents (Trio design), in order to searched for candidate monogenic inborn error of immunity (IEI) related to HSE, with a focus on rare *de novo* or biallelic variants. This resulted in the identification of compound-heterozygous predicted to be loss-of-function (pLOF) mutations of *RIPK3* in the patient. We then carried out molecular characterization of the two mutant RIPK3 proteins, by studying their expression and function following the transient transfection of HeLa and HEK293T cells or the stable transduction of HT29 cells. Finally, in order to evaluate the causality and mechanism of RIPK3 deficiency in HSE pathogenesis, we studied the known antiviral type I IFN inducing pathways, the RIPK3-dependent necroptotic and apoptotic cell death, as well as HSV-1 replication levels in the patient’s fibroblasts and human pluripotent stem cell (hPSC)-derived cortical neurons, in comparison with those from healthy controls and previously published patients with recessive deficiencies of TLR3 (21) or IFNAR1 (28) also predisposing to HSE.

### Human subjects

Informed consent was obtained in France, in accordance with local regulations and a human subjects research protocol approved by the institutional review board (IRB) of the *Institut National de la Santé et de la Recherche Médicale* (INSERM). Experiments were conducted in the United States and France, in accordance with local regulations and with the approval of the IRB of The Rockefeller University and INSERM, respectively. Approval was obtained from the appropriate French Ethics Committee (*Comité de Protection des Personnes*), the French National Agency for Medicine and Health Product Safety, INSERM in Paris, France (protocol no. C10–13), and the Rockefeller University Institutional Review Board in New York, USA (protocol no. SHZ-0676).

### Cell culture and transfection

Primary human fibroblasts were obtained from skin biopsy specimens from controls and P1, and were cultured in DMEM (GIBCO BRL, Invitrogen) supplemented with 10% fetal calf serum (FCS) (GIBCO BRL, Invitrogen). Immortalized SV40-transformed fibroblast cell lines (SV40-F) and Epstein-Barr virus (EBV)-transformed B-cell lines (EBV-B) were generated as previously described (33). More technical details are provided in Supplementary Materials. HEK293T, HeLa and HT29 cells (ATCC) were maintained in DMEM supplemented with 10% FCS. SV40-F, HEK293T and HeLa cells were transiently transfected with the aid of X-tremegen 9 DNA Transfection Reagent (XTG9-RO, Roche). RIPK3 KO cell lines were transduced with a lentiviral system, with a mock vector (Luci), or with WT and mutant RIPK3 constructs, and were then cultured under puromycin selection.

### Whole-exome and Sanger sequencing

Genomic DNA was isolated from peripheral blood cells or primary fibroblasts from the patient. Whoe exome sequencing (WES) was performed as previously described (34). More technical details are provided in Supplementary Materials. For the Sanger sequencing of the *RIPK3* variants, 300–600-bp genomic regions encompassing the mutation were amplified by PCR with site-specific oligonucleotides. PCR products were purified by ultracentrifugation through Sephadex G-50 Superfine resin (Amersham-Pharmacia-Biotech), and sequenced with the Big Dye Terminator Cycle Sequencing Kit on an ABI Prism 3700 apparatus (Applied Biosystems). SnapGene was used for sequence analysis.

### Western blots and protein immunoprecipitation (IP)

For Western blots, HEK293T, HT29 or SV40-F cell pellets were harvested, washed with PBS and lysed in RIPA buffer supplemented with cOmplete protease inhibitor cocktail (Roche). Total cell lysates were harvested. Equal amounts of protein from each sample were subjected to SDS-PAGE, and the proteins were blotted onto polyvinylidene difluoride membranes (Bio-Rad). The membranes were then probed with the desired primary antibody followed by the appropriate secondary antibody. More technical details are provided in Supplementary Materials. For co-immunoprecipitation assays, HEK293T cells were cotransfected with RIPK3 and/or RIPK1 plasmids with FLAG or Myc tags. The cells were harvested 48 h later, stored at °20°C overnight, then processed to immunoprecipitation (IP) followed by Western blotting. More technical details are provided in Supplementary Materials.

### Reverse transcription-quantitative PCR (RT-qPCR)

Total RNA was extracted with the RNeasy mini kit (Qiagen) from HEK293T cells, primary fibroblasts, SV40-F, EBV-B cells and hPSC-derived cortical neurons. The RNA was reverse-transcribed with the SuperScript III First-Strand Synthesis System (Thermo Fisher Scientific, #180800051). Reverse transcription-quantitative PCR (RT-qPCR) was performed with Applied Biosystems 2 × universal Taqman reaction mixture and Assays-on-Demand probe/primer combinations, in an ABI PRISM 7700 Sequence Detection System. The human β-glucuronidase (GUSB) gene was used for normalization with the VIC^™^/TAMRA^™^ probe (Thermo Fisher Scientific, 4310888E). The Hs01011171_g1 (RIPK3 exons 2–3) and Hs01011177_g1 (RIPK3 exons 9–10) probes were used. The other probes used in this study have been reported elsewhere (34). Results are expressed according to the ΔΔCt method, as described by the manufacturer.

### Induction of necroptosis and apoptosis

For the detection of MLKL phosphorylation, necroptosis was induced by treatment with PBZ complex (25 μg/ml poly(I:C) (Tocris, #4287); 1 μM BV6 (APExBIO, B4653); 20 μM z-VAD-fmk(APExBIO, A1902)) or TBZ complex (1000 units/ml, recombinant human TNF-alfa (TNF) (R&D Systems, 210-TA-020); 1 μM BV6; 20 μM z-VAD-fmk) for 4 h. Whole-cell lysates were collected and used for western blotting. For the detection of caspase 3 cleavage, apoptosis was induced by combined PB (25 μg/ml poly(I:C) and 1 μM BV6) or TB (1000 units/ml TNF and 1 μM BV6) treatment for various times. Whole-cell lysates were collected and western blotting was performed.

### Cell viability assay

Cells (5,000 cells/well) were used to seed Corning 96-well tissue culture plates; 16–24 h post-seeding, cells were treated with the indicated reagents, with levels of the solvent, DMSO, kept constant for all experiments. We used 25 μg/ml poly(I:C), 30 ng/ml TNF, 2 μg/ml LPS, 100 μg/ml cycloheximide (CHX), 1 μM BV6, and 20 μM Z-VAD-fmk. HSV-1 infection-induced DAI-dependent necroptotic cell death was triggered, as previously described (51). Briefly, DAI was stably overexpressed in HT29 cells with a retrovirus system, with selection on 500 μg/ml hygromycin. The cells were then infected with the F strain of HSV-1 F*mut*RHIM (MOI=5) for 24 h. Cell viability was assessed by measuring intracellular ATP levels with the CellTiter-Glo luminescent cell viability assay kit (Promega, G7571) according to the manufacturer’s instructions.

### Bulk RNA sequencing (RNAseq)

RNA was extracted from primary fibroblasts with the Quick-RNA MicroPre Kit (#R1051, Zymo Research). RNA-Seq libraries were prepared with the Illumina RiboZero TruSeq Stranded Total RNA Library Prep Kit (Illumina) and RNA-sequencing was performed on the Illumina NovaSeq platform, with a read length of 100 bp and a read depth of 40 million reads. All samples were sequenced in technical duplicates. All FASTQ files passed quality control (QC) and were aligned with the GRCh38 reference genome with STAR (2.6.1d). Gene-level features were quantified with featureCounts v1.6.0 based on GRCh38 gene annotation. Count data were normalized through ‘cpm’ (counts per million) in the EdgeR package (93), dimension-reduced through principal component analysis (PCA), and subjected to heatmap analysis with “ComplexHeatmap” (94). Differential expression (DE) analysis was performed with DESeq2 (95).

### Cell stimulation and cytokine production in a multiplex assay

We used a synthetic analog of dsRNA, poly(I:C), as a nonspecific agonist of TLR3 and MDA5/RIG-I. SV40-fibroblast cells were activated in 24-well plates, at a density of 100,000 cells/well, by incubation for 24 h with poly(I:C) at concentrations of 1, 5 and 25 μg/mL. Cells were stimulated with 25 μg/ml poly(I:C) in the presence of Lipofectamine 2000 to activate MDA5/RIG-I signaling. After 24 h, cell supernatants were used for the LEGENDplex multiplex bead assay (BioLegend, #740003 and #740984), then analyzed by flow cytometry on an Attune NxT Flow Cytometer, according to the manufacturer’s instructions. Data were analyzed with LEGENDplex Cloud-based Data Analysis Software (BioLegend), and presented in raw values and heatmaps. The heatmaps are generated using relative values for each sample as normalized to the maximum range of production levels of each cytokine among all samples (X-Min)/(Max-Min). Min: minimum production level; Max: maximum production level; X: the production level of a given cytokine in a given sample (96).

### Luciferase reporter assays

HEK293T cells (2.5 × 10^5^ cells ml^−1^) were used to seed a 96-well plate. They were transfected, in triplicate, the following day, with WT and mutant RIPK3 plasmids along with 100 ng NF-κB promoter-firefly luciferase reporter plasmid and 100 ng of *Renilla* luciferase reporter plasmid per well, in the presence of X-tremegen 9 DNA Transfection Reagent (Roche). Luciferase activities were assessed 24 h later, in a dual luciferase assay (Promega, E2940). Firefly luciferase activities were normalized against *Renilla* luciferase activities.

### Mass cytometry on fresh whole blood

Whole-blood mass cytometry was performed on 250 μl of fresh blood with a custom-designed panel (Table S4), according to Fluidigm recommendations. Labeled cells were frozen at °80°C after overnight dead-cell staining, and acquisition was performed on a Helios machine (Fluidigm). Data analysis was performed with OMIQ software.

### Gene editing with CRISPR-Cas9

Gene-editing experiments were performed as previously described (97). Briefly, guide RNA sequences were generated with the CRISPR design tool (http://crispr.mit.edu/). Two gRNAs were selected: 5’-GTCGTCGGCAAAGGCGGGTT-3’ and 5’-GCAGTGTTCCGGGCGCAACAT-3’. Forward and reverse oligonucleotides for the gRNAs were then inserted into the MLM3636 vector (a gift from K. Joung, Addgene, no. 43860). The activity of the gRNAs was assessed in HEK293T cells. We then transfected two million H9 human embryonic stem cells (hESCs) with 20 μg Cas9-GFP plasmid and 5 μg gRNA plasmid mixed in electroporation buffer (BTX, no.45–0805). Cells were sorted by FACS, on the basis of GFP signals, 48 hours after electroporation. We replated 50,000 cells with a moderate GFP fluorescence intensity and cultured them for 72 h. The cells were then detached with Accutase, counted and plated at clonal density in 96-well plates. Ten days later, the colonies were passaged and amplified. Genomic DNA was then extracted from each single-cell clone and genotyping was performed by Sanger sequencing with the following primers: Forward: 5’-AGAGGCGCCTATAAGGGAAGT-3’ and Reverse: 5’-TACACTCCAGGAGAGAGCTGG-3’.

### TOPO cloning and sequencing of cDNAs from the patient’s cells

Total RNA was extracted with the RNeasy mini kit (Qiagen) from SV40-F and hPSC-derived cortical neurons. The RNA was reverse-transcribed with the SuperScript III First-Strand Synthesis System (Thermo Fisher Scientific, #18080051) according to the manufacturer’s instructions. PCR was performed with 2 × Taq PCR master mix (APExBIO, K1034) and the following primers: Forward: 5’-CCCAGACTCCAGAGACCTCA-3’ and Reverse: 5’-AGGGGTGGCACTCTTCCTTA-3’). PCR products were inserted into the pCR2.1-TOPO vector (Life Technologies) and used to transform Steller competent cells. We picked at least 100 colonies per subject, for P1 and a healthy control. Finally, we performed PCR on these colonies and sequenced them with the following primers: Forward: 5´-GTAAAACGACGGCCAG-3´ and Reverse: 5´-CAGGAAACAGCTATGAC-3´.

### Human pluripotent stem cell (hPSC) culture and cortical neuron differentiation

Patient-specific induced pluripotent stem cells (iPSCs) were obtained by reprogramming the patients’ primary fibroblasts by infection with the non-integrating CytoTune Sendai viral vector kit (Life Technologies, USA). HESC or iPSC (together referred to as hPSC) cultures were maintained in Essential 8 medium (Life Technologies, A1517001). We used one healthy control hESC line (H9) and one healthy control iPSC line (BJ1) in this study. All hPSCs were karyotyped to ensure that the genome was intact. Patient-specific *RIPK3* mutations were confirmed by the Sanger sequencing of genomic DNA extracted from the patient’s iPSC lines. Cortical neuron differentiation was performed with hPSCs cultured in E8 essential medium in 10 cm VTN-N (Thermo Fisher Scientific)-coated plates. Cells were maintained at 37℃, under an atmosphere containing 5% CO_2_. hPSCs were differentiated with a previously described protocol (32).

### Viral infections and the quantification of viral replication

For WT HSV-1 (KOS strain, ATCC, VR-1493) infection, 5 × 10^4^ SV40-F or 1.75 × 10^5^ cortical neurons per well were used to seed 48-well plates and were infected at a MOI of 0.001 in DMEM supplemented with 2% FCS (for fibroblasts), or in neuron culture medium (for neurons). After 2 h, the cells were washed and transferred to 250 μl of fresh medium. Both cells and supernatants were collected at various timepoints and frozen. HSV-1 titers were determined by calculating the TCID_50_ ml^–1^, as previously described (34). For VSV, MeV, IAV and EMCV infections, 5 × 10^4^ SV40-F per well were added to 48-well plates in DMEM supplemented with 10% FCS. Cells were infected with VSV, MeV, IAV or EMCV at different MOI. Cells and supernatants were obtained at various timepoints and frozen, and virus levels determined by calculating the TCID50 ml^−1^ or by viral RNA quantification, as previously described (32, 81, 98). More technical details are provided in Supplementary Materials.

### Statistical analysis

When applicable, results are presented as means ± SD or means ± SEM. Mean values were compared between control cells and mutant cells by Paired t test or one-way ANOVA followed by Tukey tests for multiple comparisons. When appropriate, linear mixed models were used for log-transformed relative values, to account for repeated measurements. Statistical analysis was performed in GraphPad Prism9 (Version 9.1.1). Statistical significance is indicated as follows: ns, *P* > 0.05; *, *P* < 0.05; and **, *P* < 0.01, ***, *P* < 0.001, ****, *P* < 0.0001 in the corresponding figures.

## Data and materials availability:

The materials and reagents used are commercially available and nonproprietary, with the exception of the gene-KO or patient-specific cell lines generated by this study. The cell lines generated by this study are available from S.-Y.Z. and J.-L.C upon request under MTAs from the Rockefeller University and the Imagine Institute. The RNA sequencing data generated by this study are available in the NCBI database under the NCBI-SRA project PRJNA937264. All other data needed to support the conclusions of the paper are in the paper or the supplementary materials.

## Supplementary Material

MDAR checklist

data file S1

main supplementary
