# Supplementary material for: Encephalitis and poor neuronal death-mediated control of herpes simplex virus in human inherited RIPK3 deficiency
Source: Sci Immunol. Author manuscript; Available in PMC 2023 Jul 21. (PMC10337828; doi:10.1126/sciimmunol.ade2860)
Supplement: main supplementary [file NIHMS1911516-supplement-main_supplementary.docx]

**Supplementary Materials**

**Supplementary clinical report**

The patient (P1) was a girl born in France to non-consanguineous parents. She presented herpes simplex encephalitis (HSE) at six months of age, having previously been healthy. She had received all the recommended vaccinations. She presented with fever, a brief episode of loss of consciousness with hypotonia and an episode of right hemi-body seizure of 10 minutes’ duration. Lumbar puncture revealed a high protein content (0.45 g/l) and high levels of lymphocytes (53/mm^3^), mostly lymphocytes. PCR for herpes simplex virus (HSV1/2) was positive and CSF interferon levels were high (50 IU). Electroencephalogram (EEG) showed a left centrotemporal slowness, without paroxysmal elements. Brain magnetic resonance imaging (MRI) showed bilateral T2 hypersignal of the insulae. P1 was treated for 10 days with intravenous acyclovir and discharged following a favorable clinical course. However, seizures, hypotonia and dysmetria were observed during one year of follow-up, and were considered to be neurological sequelae of HSE.

One year later, the patient was readmitted to hospital for another episode of HSE. She presented with a high fever, seizures and an abnormal level of consciousness. Lumbar puncture showed a high protein content (0.57 g/l) and a high white blood-cell count (45/mm^3^). PCR for herpes simplex virus (HSV1/2) was again positive. A CT scan showed bilateral frontal hypodense regions, predominantly in the right hemisphere, with hemorrhagic foci. P1 was treated with intravenous acyclovir and admitted to the intensive care unit (ICU). During hospitalization, she presented persistent seizures, hypotonia and dysmetria. After three weeks of acyclovir treatment, she was discharged from the hospital.

One month later, she was again hospitalized, for another episode of encephalitis. She presented with altered levels of consciousness, dysphagia and abnormal ventilation. Lumbar puncture showed high protein levels (1.32 g/l), with 10 white blood cells/mm^3^, but PCR for HSV1/2 was negative. A brain CT-scan showed large necrotizing lesions in both frontal lobes and insulae, both parietal lobes, the right occipital lobe and basal ganglia. EEG was suggestive of a reactive coma. She was treated with high-dose intravenous steroids and acyclovir. A few days later she developed movement disorders with dyskinesia and a decrease in the level of consciousness. Based on these findings, auto-immune encephalitis was strongly suspected. P1 was treated with phenytoin (Dilantin) and then with valproate (Depakene). She was discharged from the hospital about two weeks later with severe neurological sequelae. Twenty years later, she still has multiple disabilities, with a modified Rankin score of 5/6 (severe disability; bedridden, incontinent and requiring constant nursing care and attention).

**Supplementary Materials and methods**

**Cell culture and transfection**

Primary human fibroblasts were obtained from skin biopsy specimens from controls and P1, and were cultured in DMEM (GIBCO BRL, Invitrogen) supplemented with 10% fetal calf serum (FCS) (GIBCO BRL, Invitrogen). Immortalized SV40-transformed fibroblast cell lines (SV40-F) and Epstein-Barr virus (EBV)-transformed B-cell lines (EBV-B) were generated as previously described *(33)*. Briefly, for SV40-F, about five million primary fibroblasts were transfected, by electroporation, with 4 μg of a plasmid containing T-antigen DNA. The cell suspension was then split equally between two fresh 75 cm^2^ flasks, each containing 12 ml DMEM (GIBCO BRL, Invitrogen) supplemented with 10% FCS (GIBCO BRL, Invitrogen). SV40-F clones appeared after about 15 days and were cultured and passaged for experimental use. For EBV-B cells, fresh blood samples were obtained from controls and P1, and peripheral blood mononuclear cells (PBMC) were isolated on Ficoll-Paque PLUS (GE Healthcare, USA). PBMCs were then infected with EBV and kept in culture in RPMI medium (GIBCO BRL, Invitrogen) supplemented with 10% FCS (GIBCO BRL, Invitrogen). EBV-B clones appeared in about 15 days and were cultured and passaged for experimental use.

**Whole-exome sequencing**

Genomic DNA was isolated by phenol-chloroform extraction from peripheral blood cells or primary fibroblasts from the patient. DNA (3 μg) was sheared with a Covaris S2 Ultrasonicator (Covaris). An adapter-ligated library was prepared with the TruSeq DNA Sample Prep Kit (Illumina). Exome capture was performed with the SureSelect Human All Exon 50 Mb kit (Agilent Technologies). Paired-end sequencing was performed on an Illumina HiSeq 2000 (Illumina), generating 100-base reads. The sequences were aligned with the reference human genome sequence (hg19 build), with the Burrows-Wheeler Aligner. Downstream processing was performed with the Genome Analysis Toolkit (GATK), SAMtools, and Picard Tools ([http://picard.sourceforge.net](http://picard.sourceforge.net/)). Substitution and indel calls were made with GATK Unified Genotyper and GATK IndelGenotyperV2, respectively. All calls with a Phred-scaled single nucleotide polymorphism quality ≤20 and a read coverage ≤2 were filtered out. All variants were annotated with annotation software developed in-house.

**Western blots and protein immunoprecipitation (IP)**

Cell pellets were washed with PBS and lysed in RIPA buffer supplemented with cOmplete protease inhibitor cocktail (Roche). Total cell lysates were harvested and protein concentration was determined with a BCA kit (Thermo Fisher Scientific). Equal amounts of protein from each sample were separated by SDS-PAGE and transferred onto polyvinylidene difluoride (PVDF) membranes (Millipore Sigma). The membranes were blocked by incubation with 5% skim milk and incubated with primary antibodies overnight at 4°C. Primary antibodies against the following proteins were used in this study: RIPK3 (Santa Cruz Biotechnology, sc-374639, RRID:AB_10992232), RIPK3 (Cell Signaling Technology, #13526, RRID:AB_2687467), p-RIPK3 (Cell Signaling Technology, #93654, RRID:AB_2800206), MLKL (GeneTex, GTX107538, RRID:AB_2037439), p-MLKL (Abcam, ab187091, RRID:AB_2619685), caspase 3 (Cell Signaling Technology, #9662, RRID:AB_331439), IRF3 (Proteintech, 66670-1-Ig, RRID:AB_2882024), p-IRF3 (Cell Signaling Technology, #4947, RRID:AB_823547), FLAG (Sigma-Aldrich, A8592, RRID:AB_439702), Myc (Cell Signaling Technology, #2040, RRID:AB_2148465), P65 (Santa Cruz Biotechnology, sc-8008, RRID:AB_628017), p-P65 (Cell Signaling Technology, #3033, RRID:AB_331284), ERK1/2 (Cell Signaling Technology, #4695, RRID:AB_390779), p-ERK1/2 (Cell Signaling Technology, #4370, RRID:AB_2315112), JNK1/2 (Cell Signaling Technology, #9252, RRID:AB_2250373), and p-JNK1/2 (Cell Signaling Technology, #4668, RRID:AB_823588). The membranes were then washed with PBST and incubated with the corresponding horseradish peroxidase (HRP)-conjugated secondary antibodies at room temperature for 1 h. Binding was detected by incubation with Pierce ECL western blotting substrate (Thermo Fisher Scientific). Membranes were stripped and reprobed with HRP-conjugated anti-GAPDH antibody (Proteintech, HRP-60004, RRID:AB_2737588), to control for protein loading.

For co-immunoprecipitation assays, a six-well plate was seeded with 1 million HEK293T cells by overnight incubation, and these cells were cotransfected with RIPK3 and/or RIPK1 plasmids with FLAG or Myc tags. The cells were harvested 48 h later, and washed with ice-cold PBS. The cell pellets were stored at -20°C overnight. The frozen cell pellets were lysed with IP buffer supplemented with an EDTA-free protease inhibitor cocktail (Roche, #11836170001). Whole-cell lysates were then incubated overnight at 4°C with EZview™ Red ANTI-FLAG® M2 Affinity Gel (Millipore, F2426) or Anti-c-Myc Agarose Affinity Gel (Millipore, A7470). Immunoprecipitates were washed three times with IP buffer and eluted with 1% SDS protein loading buffer at 95°C for 10 min. Western blotting was performed with FLAG M2 peroxidase (HRP) antibody (Sigma-Aldrich, A8592) and Myc-tag (9B11) mouse mAb (HRP Conjugate) (Cell Signaling Technology, #2040). Membranes were stripped with stripping buffer (Thermo Fisher Scientific, PI21063), blocked and reprobed with HRP-conjugated anti-GAPDH antibody (Proteintech, HRP-60004, RRID:AB_2737588), to control for whole-cell lysate protein loading.

**Immunostaining**

HeLa cells were transiently transfected with WT or mutant RIPK3 in the presence of X-tremeGENE9 transfection reagent (Roche, # 06365787001). After 48 h of incubation, cells were fixed by incubation with 4% paraformaldehyde for 15 min at 37°C, washed three times with PBS, permeabilized with 0.1% Triton and blocked by incubation with 6% donkey serum in 0.1% Triton for 1 h. The cell cultures were stained by incubation overnight with the anti-RIPK3 antibody (Santa Cruz Biotechnology, sc-374639, RRID:AB_10992232). Cells were washed three times with PBS and incubated with an anti-mouse Alexa 488 secondary antibody (Life Technologies, A-10680). Cells were washed three times with PBS and mounted in DAPI-containing Prolong Gold mounting medium (Thermo Fisher Scientific, #62248). Slides were examined with a confocal laser microscope (Confocal Leica SP8 gSTED). All images were acquired with APO CS2 63×/1.4 oil objectives. Images were exported as tif files and image analysis was performed with ImageJ software. For each independent experiment, we analyzed 5-12 cells for each set of conditions. Each field was selected on the basis of DAPI-positive staining, which was used to define the nuclear region (NR).

**Viral infections and the quantification of viral replication**

# For VSV and MeV infections, 5 × 10^4^ SV40-F per well were added to 48-well plates in DMEM supplemented with 10% FCS. Cells were infected with MeV (MOI = 0.5) or VSV (MOI = 0.1, Indiana strain, gift from Pierre Lebon’s laboratory) in DMEM supplemented with 2% FCS for 2 h, washed twice with PBS and then cultured in DMEM supplemented with 2% FCS. Cells and supernatants were obtained at various timepoints and frozen. Viral titers were determined by calculating the TCID50 ml^-1^, as previously described *(34)*.

For IAV infection, 5 × 10^4^ SV40-F per well were added to 48-well plates and infected with IAV (A/California/4/2009 strain) at a MOI of 10 as previously described *(81)*.

For EMCV infection, 5 × 10^4^ SV40-F per well were added to 48-well plates and infected with EMCV (gift from Pierre Lebon’s Laboratory) at a MOI of 0.01 in DMEM supplemented with 2% FCS for 1 h, washed twice in PBS, and then cultured in DMEM supplemented with 2% FCS. The mixture of cells and supernatant was collected at various time points and frozen for total RNA extraction with the Quick-RNA MicroPre Kit (#R1051, Zymo Research) according to the manufacturer’s protocol. Total RNA was reverse-transcribed with random hexamers and the SuperScript III First-Strand Synthesis System (#18080051, Thermo Fisher Scientific) according to the manufacturer’s instructions. EMCV genome copy number was determined with SYBR Green qPCR methods (#4385612, Applied Biosystems) for EMCV 3D, with the primers 5’-GACGCTTGAAGACGTTGTCTTCTTA-3’ and 5’-CCCTACCTCACGGAATGGGGCAAAG3’, as previous described *(32, 98)*. Meanwhile, the primers 5’-CACCAGGATCCACCTCTGAT-3’ and 5’-TCCAAATGAGCTCTCCAACC-3’ were used for β-glucuronidase (GUSB), the housekeeping gene for normalization. The results are expressed according to the ΔΔCt method, as recommended by the kit manufacturer.

**Cortical neuron differentiation from human pluripotent stem cell (hPSC)**

For cortial neuron differentiation, hPSCs were dissociated with Accutase to obtain a single-cell suspension, which was plated at a density of 300,000 cells/cm^2^ on Matrigel-coated plates, in Essential 8 medium supplemented with 10 μM ROCK inhibitor (Tocris, Y-27632, #1254). Cells were cultured in Essential 6 medium supplemented with 100 nM LDN193189 (Stemgent, #04-0074) and 10 μM SB431542 (STEMCELL Technologies, #72234) for 10 days, with 2 μM XAV939 (Tocris, #3748/10) added for the first three days of differentiation. For days 11-20 of differentiation, cells were cultured in N2 medium with 1:1000 B27 Supplement (Life Technologies, #12587-010) to promote the development of neural progenitor cells (NPCs). NPCs were then dissociated and re-plated on poly-ornithine/fibronectin/laminin-coated plates. They were maintained in neurobasal medium with DAPT gamma-secretase inhibitor (R&D Systems, #2634), recombinant human BDNF (R&D Systems, 248-BD), L-ascorbic acid (Millipore Sigma, A4034), recombinant human GDNF (PeproTech, #450-10), cyclic AMP sodium (dbcAMP) (SigmaAldrich, D0627), L-glutamine (Life Technologies, #25030024), and B27 supplement (Life Technologies, #12587-010) to promote neuronal differentiation and maturation. All experiments were performed on hPSC-derived cortical neurons at DIV 50.

**
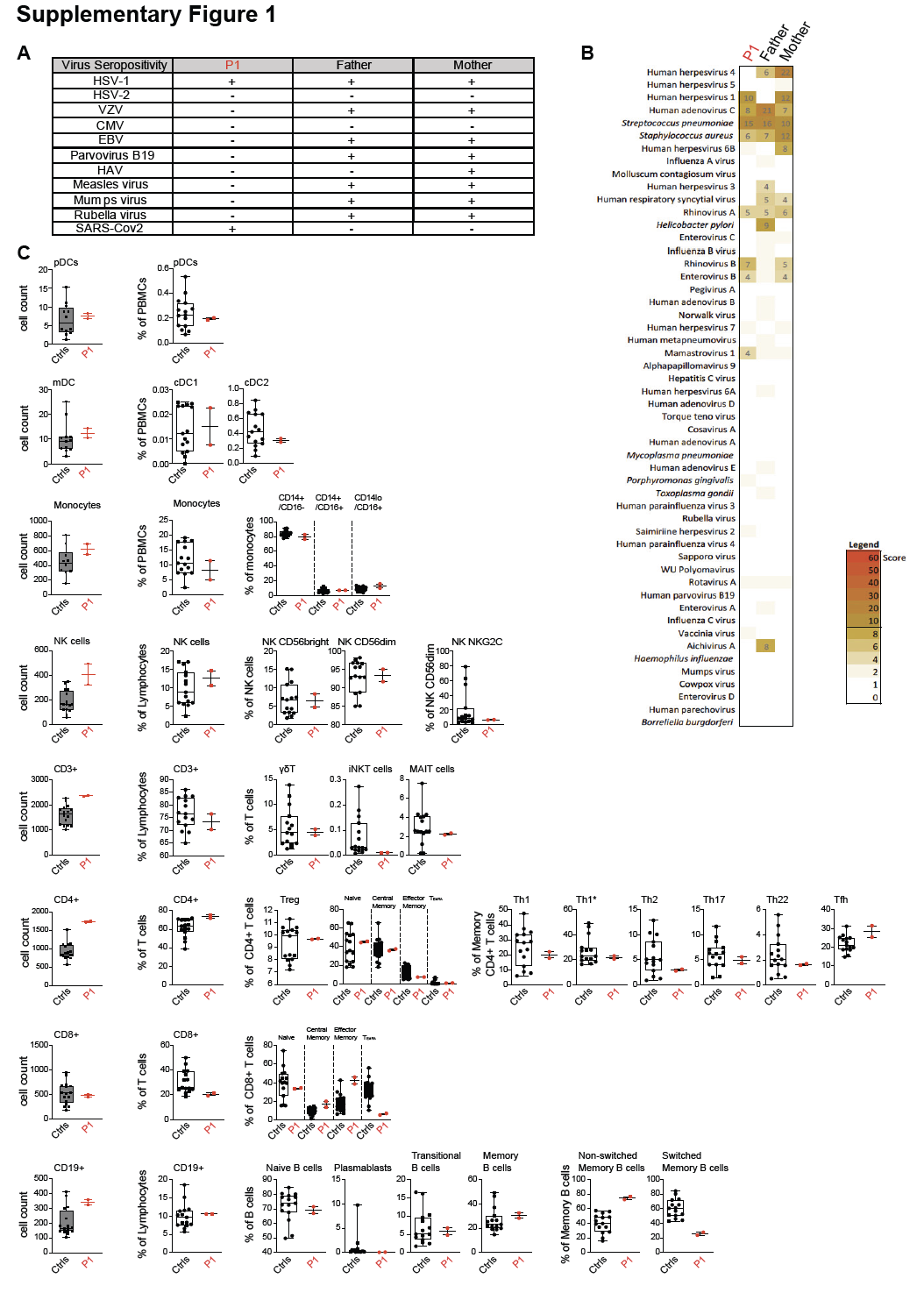
**


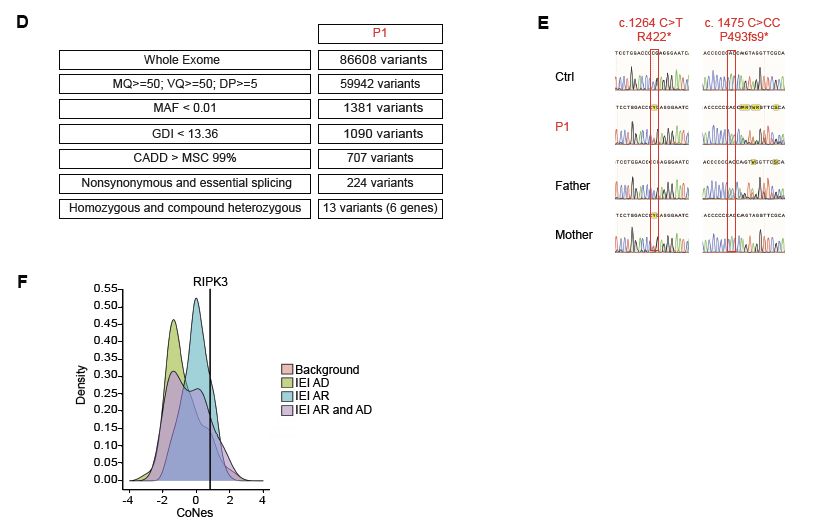


**Figure S1. Compound heterozygous RIPK3 mutations in a patient with HSE, related to Figure 1**

**A.** Viral serological test on P1 and her parents. **B.** VirScan test on P1 and her parents. **C.** Deep immunophenotyping by mass cytometry (CyTOF) with PBMCs from P1 and healthy donors (Ctrls). **D.** Filtering criteria for variants from the whole-exome sequencing of P1. E. Electropherogram showing the mutations in exon 8 and exon 10 of RIPK3 in P1 and her parents. **F.** CoNes analysis of negative selection for RIPK3.


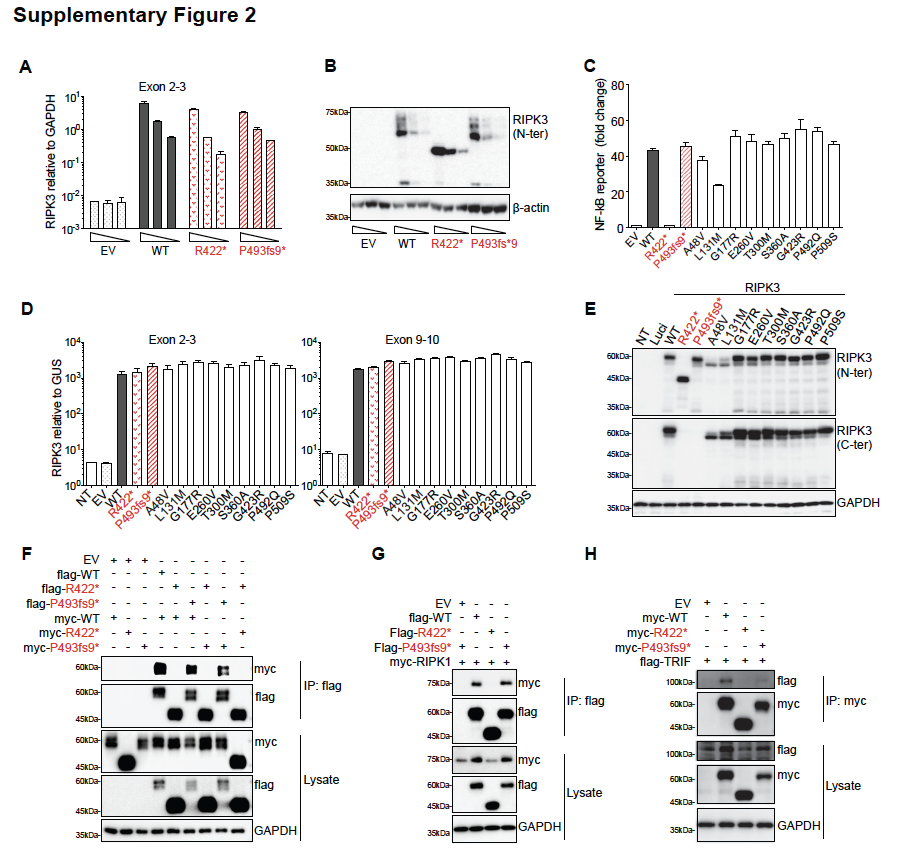


**Figure S2. In vitro production and function of the RIPK3 variants after transient transfection, related to Figure 2**

**A.** *RIPK3* mRNA levels were measured by RT-qPCR in HEK293T cells transfected with an empty vector (EV), or WT and mutant RIPK3 constructs at various doses for 24 h. The data shown are the means of three biological replicates from one experiment. **B.** Immunoblot analysis of RIPK3 levels in HEK293T cells, as in (**A**). The results shown are representative of three independent experiments. **C.** RIPK3 overexpression-mediated NF-κB promoter-driven reporter assay in HEK293T cells transfected with the NF-κB reporter plasmid, along with various doses of empty vector (EV), WT and patient-specific mutant RIPK3, and homozygous nonsynonymous RIPK3 variants from the gnomAD database. The data shown are the means of three biological replicates from one experiment, representative of four independent experiments. **D.** *RIPK3* mRNA levels were determined by RT-qPCR in HEK293T cells transfected as in (**C**). We used two probes, targeting exons 2-3 (left) and exons 9-10 (right) of RIPK3. The data shown are the means of three biological replicates from three independent experiments. **E.** RIPK3 protein levels were determined by western blotting in HEK293T cells transfected as in (**C**). The RIPK3 protein was detected with antibodies against the N-terminus (N-ter) or C-terminus (C-ter) of RIPK3. The results shown are representative of three independent experiments. **F.** FLAG-tagged WT and mutant RIPK3 constructs were co-expressed with Myc-tagged WT and mutant RIPK3 constructs in HEK293T cells, which were then subjected to immunoprecipitation (IP) with anti-FLAG antibody-conjugated agarose beads, and immunoblotting with anti-FLAG or anti-Myc antibodies. The results shown are representative of three independent experiments. **G.** FLAG-tagged WT and mutant RIPK3 constructs were co-expressed with Myc-tagged WT RIPK1 constructs in HEK293T cells, which were then subjected to IP and immunoblotting as in (**F**). The results shown are representative of three independent experiments. **H.** Myc-tagged WT and mutant RIPK3 constructs were co-expressed with FLAG-tagged WT TRIF constructs in HEK293T cells, which were then subjected to IP and immunoblotting as in (**F**). The results shown are representative of two independent experiments.


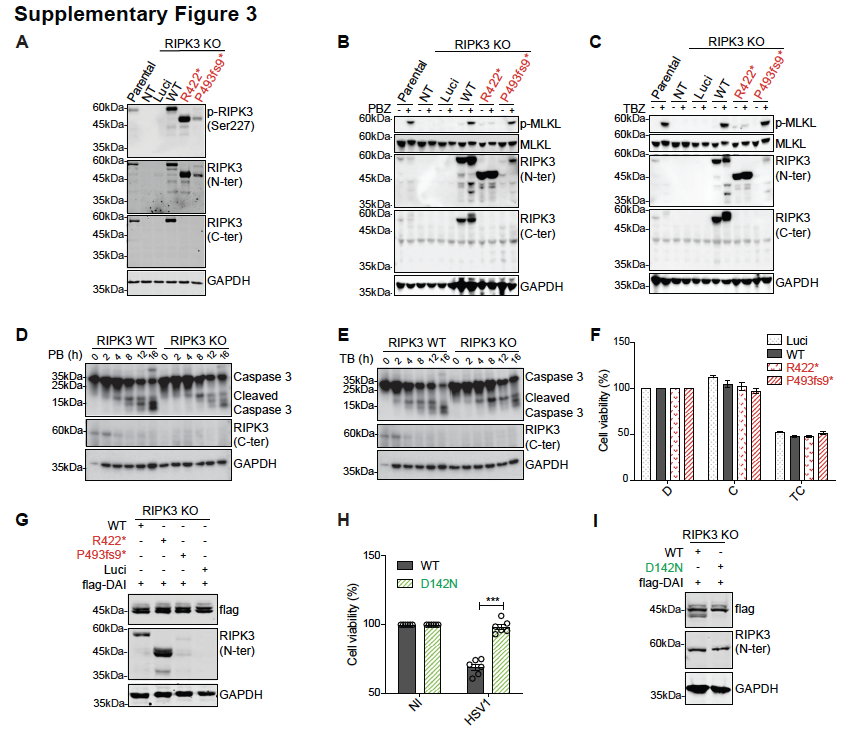


**Figure S3. In vitro production and function of the RIPK3 mutants after stable transduction, related to Figure 3**

**A.** Immunoblotting analysis of total RIPK3 and autophosphorylated RIPK3 protein levels in the parental HT29 cells, and in RIPK3 KO HT29 cells left non-transfected (NT) or stably transfected with a mock vector (Luci) or with WT or mutant RIPK3 constructs in a lentiviral system. The results shown are representative of two independent experiments. **B-C.** Immunoblot analysis of phosphorylated MLKL (p-MLKL, Ser358) in parental HT29 cells, and RIPK3 knockout (KO) HT29 cells not transfected (NT), or stably expressing luciferase (Luci) or WT and mutant RIPK3, treated with DMSO solvent (D), PBZ complex containing poly(I:C), BV6 and Z-VAD (B), or TBZ complex containing TNF, BV6 and Z-VAD (C) for 4 h. The results shown are representative of three independent experiments. **D-E.** Immunoblot analysis of full-length and cleaved caspase 3 in parental HT29, and RIPK3 KO HT29 cells, treated with PB complex containing poly(I:C) and BV6 (D), or TB complex containing TNF and BV6 (E) for the times indicated. The results shown are representative of three independent experiments. **F.** Viability of RIPK3-/- HT29 cells stably expressing WT and mutant RIPK3 constructs, treated with DMSO solvent (D), or with TNF, or TC (TNF + cycloheximide) complex for the times indicated. The results shown are the means from three biological replicates from one experiment, representative of two independent experiments. **G.** Immunoblot analysis of FLAG-tagged DAI in RIPK3 KO HT29 cells stably co-expressing FLAG-DAI with a mock vector (Luci) or with WT or mutant RIPK3 constructs in a lentiviral system. The results shown are representative of two independent experiments. **H.** Viability of RIPK3-/- HT29 cells stably co-expressing FLAG-tagged DAI with WT and the kinase-dead mutant D142N RIPK3, left non-infected (NI), or after infection with HSV-1 FmutRHIM (MOI=5) for 24 h. The data shown are means ± SEM from two independent experiments, with three biological replicates per experiment. P values were obtained by Paired t test, and the corresponding P values are indicated. ***P<0.001. **I.** Immunoblotting analysis of HT29 cells stably co-expressing FLAG-DAI with WT or D142N RIPK3 constructs in a lentiviral system. The results shown are representative of two independent experiments.


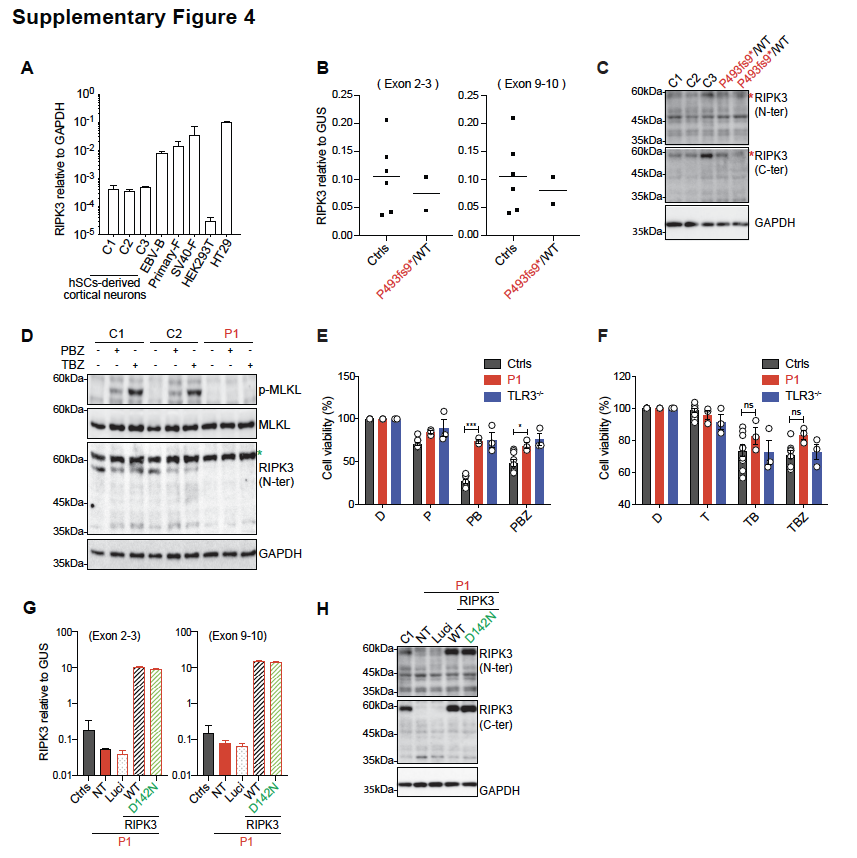


**Figure S4. Endogenous RIPK3 production in various cell types, and the impairment of TLR3- or TNFR1-mediated necroptotic signaling in P1’s fibroblasts, related to Figure 4**

**A.** *RIPK3* mRNA levels, as measured by RT-qPCR in various cell types. The data shown are the means of at least two biological replicates from one experiment. **B.** *RIPK3* mRNA levels were measured by RT-qPCR in SV40-F from healthy controls (Ctrls) and two individuals heterozygous for the P493fs9* mutation, with two probes targeting exons 2-3 (upper panel) and exons 9-10 (lower panel) of RIPK3, respectively. The data shown are the means of two biological replicates from one experiment. **C.** Immunoblot analysis of endogenous RIPK3 expression in SV40-F from the healthy controls (C1, C2, C3) and two individuals heterozygous for the P493fs9* mutation, with antibodies against the N-terminus and C-terminus of RIPK3. The red asterisks indicate the bands corresponding to RIPK3. The results shown are representative of three independent experiments. **D.** Immunoblot analysis of p-MLKL in SV40-F from healthy controls (C1, C2) and P1, stimulated with either PBZ or TBZ for 4 h. The green asterisk indicates non-specific bands. The results shown are representative of three independent experiments. **E.** Viability of primary fibroblasts from healthy controls (Ctrls, n=3), P1 and a TLR3-/- HSE patient, treated with DMSO solvent (D), or with poly(I:C), PB (poly(I:C) + BV6), or PBZ complex for the times indicated. The results shown are the means ± SEM from three independent experiments. Each dot represents the mean of three biological replicates from one independent experiment. **F.** Viability of primary fibroblasts from healthy controls, P1 and a TLR3-/- patient, treated with DMSO solvent (D), TNF, TB (TNF + BV6), or TBZ complex for the times indicated. The results shown are the means ± SEM from three independent experiments. Each dot represents the mean of three biological replicates from one independent experiment. In **E** and **F**, P values were obtained by one-way ANOVA with Tukey’s multiple comparison of P1’s cells with control cells, and the corresponding P values are indicated. ns-not significant, *P<0.05, ****P<0.0001. **G.** RIPK3 mRNA levels were measured by RT-qPCR in SV40-F from healthy controls (Ctrls) and P1, either left non-transduced or transduced with Luci, WT or D142N RIPK3 expressing lentivirus for 48 h, with two probes targeting exons 2-3 (upper panel) and exons 9-10 (lower panel) of RIPK3. The data shown are the means of three biological replicates from one experiment. **H.** Immunoblot analysis of RIPK3 expression in SV40-F from the healthy control (C1) and P1, as in (G), with antibodies against the N-terminus and C-terminus of RIPK3. The results shown are representative of three independent experiments.


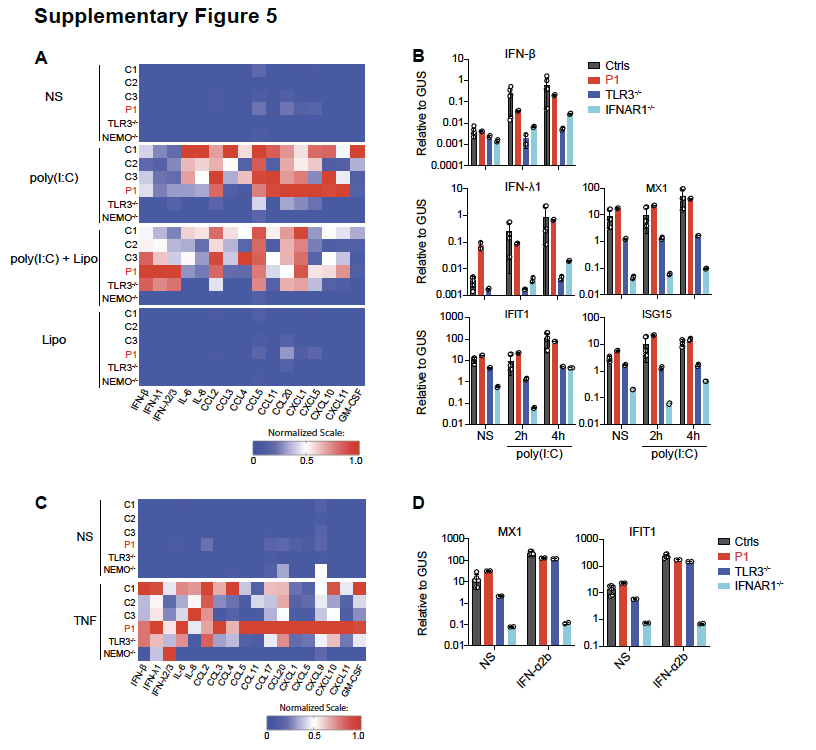


**Figure S5. Intact TLR3 and type I IFN responsiveness in P1 fibroblasts, related to Figure 5**

**A.** SV40-F from healthy controls (C1, C2, C3), P1, TLR3-/- and NEMO-/- patients were left non-stimulated (NS) or were stimulated with poly(I:C) alone, Lipofectamine alone (Lipo), or both (poly(I:C)+Lipo), for 24 h. The levels of the indicated cytokines in the culture supernatant were determined with Legendplex cytometric bead arrays. The heatmap was generated as described in the Method. Each color represents the relative concentration of a particular analyte. Blue and red indicate low and high concentrations, respectively. The data shown are the means of two independent experiments, with three biological replicates per experiment. **B.** The levels of the *IFNB*, *IFNL1*, *MX1*, *IFIT1* and I*SG15* mRNAs were determined by RT-qPCR in SV40-F from healthy controls (Ctrls, n=3), P1 and HSE patients with AR TLR3 or IFNAR1 deficiencies. The cells were left non-stimulated (NS) or were stimulated with 25 μg/mL poly(I:C) for the times indicated. The data shown are the means of two biological replicates from one experiment, representative of two independent experiments. **C.** SV40-F from healthy controls, P1 and TLR3-/- and NEMO-/- patients were left unstimulated or were stimulated with 20 ng/mL TNF for 24 h. The levels of the indicated cytokines in the culture supernatants were determined with Legendplex cytometric bead arrays and presented as in (**A**). The data shown are the means of two independent experiments, with three biological replicates per experiment. **D.** *MX1* and *IFIT1* mRNA levels were determined by RT-qPCR in SV40-F from healthy controls (n=3), P1 and HSE patients with AR TLR3 or IFNAR1 deficiencies. The cells were left non-stimulated or were stimulated with 1000 units/ml IFNα2b for 8 h. The data shown are the means of two biological replicates from two independent experiments.


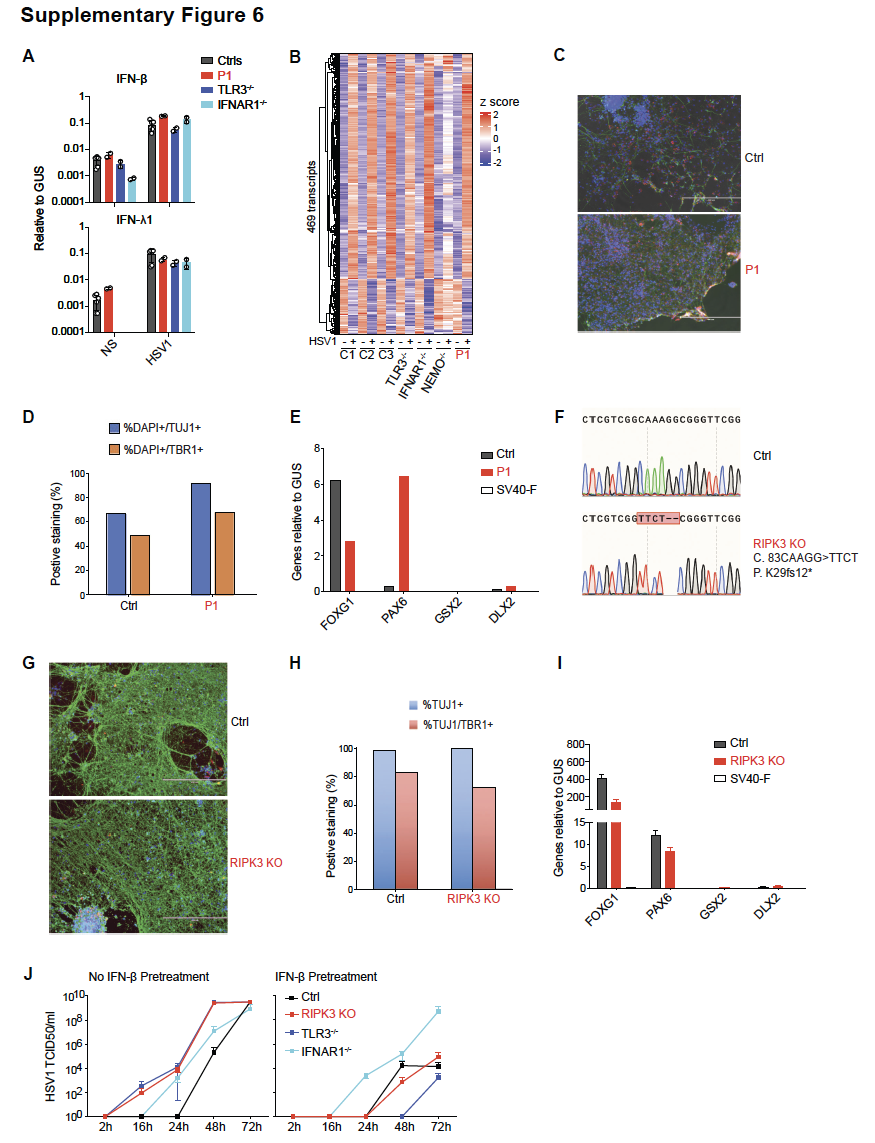


**Figure S6. Characterization of the hPSC-derived CNS cortical neurons, related to Figure 6**

**A.** *IFNB* and *IFNL1* mRNA levels were determined by RT-qPCR in SV40-F from healthy controls (n=3), P1 and HSE patients with AR TLR3 or IFNAR1 deficiencies. The cells were left uninfected (NS) or were infected with the KOS strain of HSV-1 (MOI=1) for 24 h. The data shown are the means of two biological replicates from one experiment, representative of two independent experiments. ND, not detected. **B.** Interferon-stimulated genes (ISGs) differentially expressed between HSV-1 and NS in human primary fibroblast without (NS) or with HSV-1 infection for 24 hours, in cells from healthy controls (Ctrls, n=3), P1 and other patients with recessive TLR3, IFNAR1 or NEMO deficiencies. The heatmap includes 469 known fibroblastic ISGs with relative absolute fold-changes in expression > 2 (in all three healthy controls) in response to HSV-1 relative to NS samples in the control group. **C.** Representative images of hPSC-derived cortical neurons from a healthy control (Ctrl) and P1. Cells were fixed and stained for chromatin (DAPI, blue), a neuron-specific tubulin isoform (Tuj1, green) and a cortex-specific nuclear protein (TBR1, red). **D.** Quantification of the proportion of hPSC-derived cortical neurons among total cells based on the immunostaining of cells from a healthy control and P1. **E.** *FOXG1*, *PAX6*, *GSX2* and *DLX2* mRNA levels were determined by RT-qPCR in hPSC-derived cortical neurons from a healthy control and P1. SV40-F from a healthy control were used as a negative control in this assay. The results shown are representative of three independent experiments. **F.** Histogram representation of the homozygous RIPK3 mutation introduced by CRISPR-Cas9, confirmed by Sanger sequencing on genomic DNA from the gene-edited hPSC line. Sequencing results for the parental line are also shown. **G.** Representative images of cortical neurons from the parental and RIPK3 KO hPSC lines. Cells were fixed and stained for chromatin (DAPI, blue), a neuron-specific tubulin isoform (Tuj1, green) and a cortex-specific nuclear protein (TBR1, red). **H.** Quantification of the proportion of cortical neurons among total cells based on the immunostaining of cortical neurons from parental and RIPK3 KO hPSC lines. **I.** *FOXG1*, *PAX6*, *GSX2* and *DLX2* mRNA levels were determined by RT-qPCR in cortical neurons from parental and RIPK3 KO hPSC lines. SV40-F from a healthy control were used as a negative control in this assay. The data shown are the means of at least three biological replicates from three independent experiments. **J.** hPSC-derived cortical neurons from healthy parental control cells, RIPK3 KO cells and HSE patients with AR TLR3 or IFNAR1 deficiency, with or without IFN-β pretreatment for 24 h, were infected with HSV-1 (MOI=0.001) for the times indicated. HSV-1 replication was quantified by the TCID50 virus titration method. The results shown are the means of two biological replicates from one experiment.

**Table S1. De novo nonsynonymous variants in P1**

| Gene | GDI | Annotation | Change | Zygosity | MAF (gnomAD) | CADD |
| --- | --- | --- | --- | --- | --- | --- |
| *CSMD2* | 13.095 | missense | p.Ala1821Asp | het | 0 | 29.9 |
| *VCAM1* | 4.249 | missense | p.Ser529Pro | het | 0 | 17.26 |
| *TNR* | 4.363 | missense | p.Val936Met | het | 0 | 25.2 |
| *IQCJ-SCHIP1* | 1.929 | indel-inframe | p.Ser117del | het | 0 | 21.1 |
| *NUP153* | 17.573 | missense | p.Ala605Val | het | 0 | 19.77 |
| *MUC21* | 7.824 | missense | p.Thr173Ala | het | 0.0012 | 0.001 |
| *MUC21* | 7.824 | missense | p.Thr173Ile | het | 0.0007 | 0.183 |
| *NOTCH4* | 14.382 | missense | p.Gly863Trp | het | 0 | 25.6 |
| *NACAD* | 8.227 | missense | p.Ala601Val | het | 0.0006 | 6.387 |
| *MUC6* | 15.486 | missense | p.Lys1491Met | het | 0 | 0.882 |
| *EIF3M* | 0.769 | missense | p.Leu38Pro | het | 0 | 23.2 |
| *TECTA* | 14.14 | missense | p.Ala774Val | het | 0 | 15.89 |
| *SCYL2* | 4.911 | missense | p.Glu663Lys | het | 0.0065 | 19.28 |
| *VTI1B* | 1.039 | missense | p.Arg176His | het | 0.0035 | 23.9 |
| *MUC16* | 31.774 | missense | p.Thr13162Ser | het | 0.0037 | 0.001 |
| *ZFP30* | 2.13 | missense | p.Ser210Phe | het | 0.0041 | 24 |
| *ZNF428* | 2.284 | indel-inframe | p.Glu154del | het | 0 | 15.19 |
| *UBASH3A* | 4.661 | missense | p.Arg395Ser | het | 0 | 23.3 |
| *DNAJB7* | 4.524 | missense | p.Pro145Thr | het | 0.002 | 11.57 |
| *FAM9A* | 3.499 | indel-inframe | p.Glu232del | het | 0.0038 | 3.146 |
|  |  |  |  |  |  |  |

Note: All de novo nonsynonymous or essential-splicing variations found in the exome of P1 are listed. GDI, gene damage index. MAF, minor allele frequency. CADD, combined annotation-dependent depletion score.

**Table S2. Homozygous and compound heterozygous rare nonsynonymous variants in P1**

| Gene | GDI | Annotation | Change | Zygosity | MAF (gnomAD) | CADD |
| --- | --- | --- | --- | --- | --- | --- |
| *PCDHA1* | 4.35 | missense | p.Asn449His | hom | 0.00427296 | 23.9 |
| *BCLAF1* | 11.75 | missense | p.Pro758Thr | het | 0.00797482 | 20.6 |
| *BCLAF1* | 11.75 | missense | p.Asn629Ser | het | 0.00216826 | 20.1 |
| *PABPC1* | 12.5 | missense | p.Arg475Gln | het | 0.00189407 | 24.4 |
| *PABPC1* | 12.5 | missense | p.Ile454Thr | het | 7.32E-04 | 22.7 |
| *PABPC1* | 12.5 | stop-gained | p.Glu345* | het | 0.00150455 | 42 |
| *PABPC1* | 12.5 | indel-frameshift | p.Lys157fs | het | 3.26E-05 | 32 |
| *ZFAT* | 5.76 | missense | p.Arg546Trp | het | 0.00961967 | 18.04 |
| *ZFAT* | 5.76 | missense | p.Ser470Cys | het | 0.00168768 | 29.3 |
| *RIPK3* | 3.83 | indel-frameshift | p.Pro493fs | het | 0.00207218 | 23.5 |
| *RIPK3* | 3.83 | stop-gained | p.Arg422* | het | 0.00105122 | 34 |
| *CDC27* | 0.49 | missense | p.Trp644Arg | het | 0.002817 | 33 |
| *CDC27* | 0.49 | missense | p.Tyr641Cys | het | 0.00702138 | 32 |

Note: homozygous or compound-heterozygous nonsynonymous or essential-splicing variations (MAF<0.01 in gnomAD, CADD>mutation significance cut-off of 95% confidence interval) were found in 1, 5 genes (GDI< 13.83), respectively, in the exome of P1.

**Table S3. Homozygous nonsynonymous *RIPK3* variants reported by gnomAD**

| Variant | Annotation | Allele Frequency | Homozygote Count | CADD |
| --- | --- | --- | --- | --- |
| p.Pro492Gln | Missense | 0.07635314 | 1229 | 18.14 |
| p.Thr300Met | Missense | 0.05053898 | 472 | 0.009 |
| **p.Pro493ThrfsTer9** | **Frameshift** | **0.00261058** | **1** | **23.5** |
| **p.Arg422Ter** | **Stop-gained** | **0.00156793** | **1** | **35** |
| p.Glu260Val | Missense | 0.00051304 | 1 | 18.24 |
| p.Ala48Val | Missense | 0.00050224 | 2 | 23.6 |
| p.Gly423Arg | Missense | 0.00042891 | 1 | 0.394 |
| p.Gly177Arg | Missense | 0.00028656 | 1 | 1.607 |
| p.Ser360Ala | Missense | 9.55E-05 | 1 | 1.465 |
| p.Leu131Met | Missense | 3.98E-05 | 1 | 24.2 |
| p.Pro509Ser | Missense | 1.59E-05 | 1 | 13.16 |

**Table S4. Antibodies used for mass cytometry on fresh whole blood**

| Metal | Target | Clone | Manufacturer | Catalog |
| --- | --- | --- | --- | --- |
| 163Dy | CXCR3 | G025H7 | Fluidigm | 3163004B |
| 152Sm | TCRgd | 11F2 | Fluidigm | 3152008B |
| 142Nd | CD19 | HIB19 | Fluidigm | 3142001B |
| 144Nd | CD38 | HIT2 | Fluidigm | 3144014B |
| 151Eu | CD123 | 6H6 | Fluidigm | 3151001B |
| 153Eu | Va7.2 | 3C10 | Fluidigm | 3153024B |
| 154Sm | CD3 | UCHT1 | Fluidigm | 3154003B |
| 155Gd | CD45RA | HI100 | Fluidigm | 3155011B |
| 158Gd | CD27 | L128 | Fluidigm | 3158010B |
| 159Tb | CD1c | L161 | Biolegend | 331502 |
| 161Dy | CLEC9A | 8F9 | Fluidigm | 3161018B |
| 164Dy | CD161 | HP-3G10 | Fluidigm | 3164009B |
| 168Er | CD8 | SK1 | Fluidigm | 3168002B |
| 170Er | iNKT | 6B11 | Fluidigm | 3170015B |
| 175Lu | CCR4 | L291H4 | Fluidigm | 3175035A |
| 174Yb | CD4 | RPA-T4 | Biolegend | 300502 |
| 162Dy | CD21 | REA940 | Miltenyi Biotec Inc. | 130-124-315 |
| 165Ho | NKG2C | REA205 | Miltenyi Biotec Inc. | 130-122-278 |
| 148Nd | CD20 | 2H7 | Biolegend | 302302 |
| 173Yb | HLA-DR | L243 | Fluidigm | 3173005B |
| 156Gd | CCR10 | REA326 | Miltenyi Biotec Inc. | 130-122-317 |
| 089Y | CD45 | HI30 | Fluidigm | 3089003B |
| 116Cd | CD66b | QA17A51 | Biolegend | 396902 |
| 141Pr | CCR6 | G034E3 | Fluidigm | 3141003A |
| 143Nd | CD127 | A019D5 | Fluidigm | 3143012B |
| 147Sm | CD11c | Bu15 | Fluidigm | 3147008B |
| 149Sm | CD25 | 2A3 | Fluidigm | 3149010B |
| 150Nd | NKVFS1 | NKVFS1 | Bio Rad | MCA2243GA |
| 167Er | CCR7 | G043H7 | Fluidigm | 3167009A |
| 169Tm | NKG2A | Z199 | Fluidigm | 3169013B |
| 171Yb | CXCR5 | RF8B2 | Fluidigm | 3171014B |
| 166Er | CD24 | ML5 | Fluidigm | 3166007B |
| 145Nd | CD31 | WM59 | Fluidigm | 3145004B |
| 160Gd | CD14 | M5E2 | Fluidigm | 3160001B |
| 176Yb | CD56 | NCAM16.2 | Fluidigm | 3176008B |
| 172Yb | CD57 | HNK-1 | Biolegend | 359602 |
| 150Nd | KIR3DL1L2 | REA970 | Miltenyi Biotec Inc. | 130-126-489 |
| 146Nd | IgD | IA6-2 | Fluidigm | 3146005B |
| 209Bi | CD16 | 3G8 | Fluidigm | 3209002B |
